# Temporal and protein-specific S-palmitoylation supports synaptic and neural network plasticity

**DOI:** 10.1007/s00018-025-05893-5

**Published:** 2025-10-11

**Authors:** Agata Pytyś, Rabia Ijaz, Anna Buszka, Jacek Miłek, Izabela Figiel, Patrycja Wardaszka-Pianka, Matylda Roszkowska, Natalia Mierzwa, Adam Wojtas, Eli Kerstein, Remigiusz Serwa, Katarzyna Kalita, Rhonda Dzakpasu, Magdalena Dziembowska, Jakub Włodarczyk, Tomasz Wójtowicz

**Affiliations:** 1 Laboratory of Cell Biophysics, Nencki Institute of Experimental Biology of the Polish Academy of Sciences, Warsaw, Poland; 2 Laboratory of Molecular Basis of Behavior, Nencki Institute of Experimental Biology of the Polish Academy of Sciences, Warsaw, Poland; 3 Department of Animal Physiology, Faculty of Biology, University of Warsaw, Warsaw, Poland; 4 Laboratory of Neurobiology, BRAINCITY, Nencki Institute of Experimental Biology of the Polish Academy of Sciences, Warsaw, Poland; 5 Department of Physics, Georgetown University, Washington, D.C USA; 6 Proteomics Core Facility, The International Institute of Molecular Mechanisms and Machines of the Polish Academy of Sciences, Warsaw, Poland

**Keywords:** Post*-*translational modifications (PTMs), Protein acylation, NMDAR-dependent LTP (long-term potentiation), Synaptoneurosomes, Acyl-biotin exchange assay, Mass spectrometry

## Abstract

**Background:**

Synaptic plasticity, a fundamental process underlying learning and memory, depends on activity-driven changes in neural connectivity. S-palmitoylation, a reversible post*-*translational lipid modification, modulates synaptic protein function by influencing protein conformation, localization, trafficking, and molecular interactions. Despite its known significance in neuronal function, the temporal and protein-specific dynamics of S-palmitoylation during synaptic plasticity remain poorly understood.

**Methodology & Principal Findings:**

Using electrophysiological methods, molecular biology, proteomics, and imaging across various models (neuronal cultures, hippocampal slices, and synaptoneurosomes), we investigated S-palmitoylation during synaptic activity. Induction of long-term potentiation (LTP) resulted in protein-specific palmitoylation changes without altering global levels. In hippocampal slices, synaptophysin and PSD95 displayed distinct temporal patterns of palmitoylation, influenced by LTP. Deacylation experiments using N-(tert*-*butyl)hydroxylamine (NtBuHA) demonstrated that protein S-palmitoylation is crucial for organizing neuronal spiking and enabling LTP, particularly in the stratum radiatum. Mass spectrometry of synaptoneurosomes revealed a palmitoylome including over 700 proteins, with stimulation-induced predominant depalmitoylation. Differentially palmitoylated proteins were associated with synaptic vesicle cycling, cytoskeletal dynamics, and neurotransmitter release. What is interesting is that synaptoneurosomes contained active palmitoylation machinery, supporting rapid, target*-*specific responses to NMDA receptor activation.

**Conclusions:**

Temporal and protein-specific S-palmitoylation emerges as a vital mechanism for synaptic plasticity, contributing to neuronal network function and memory formation. These findings elucidate how palmitoylation acts as a dynamic regulator of synaptic activity and offer insights into its regulation. The study highlights the potential of targeting palmitoylation pathways for enhancing neuronal function.

**Supplementary Information:**

The online version contains supplementary material available at 10.1007/s00018-025-05893-5.

## Introduction

A defining feature of the mammalian brain is its capacity to process and retain information within highly structured neuronal networks. Neuronal plasticity - the ability of neurons to adapt their connectivity, properties, and structure in response to activity - is fundamental to learning and memory. These processes involve the reorganization of existing synapses, modulation of synaptic efficacy, and adjustments to intrinsic neuronal excitability. Understanding the molecular mechanisms that govern these dynamic changes is central to advances in neuroscience, neuropharmacology, and medical research.

Post*-*translational modifications (PTMs) add a critical layer of regulation to the proteins driving neuronal plasticity. By modulating protein function, localization, stability, and interactions, PTMs enable the rapid and precise tuning of cellular processes necessary for synaptic remodeling and network adaptation. Among PTMs, lipid modifications have emerged as key regulators of neuronal function. These modifications, including N-myristoylation, N-acylation, O-acylation, and S-acylation, alter the hydrophobicity of proteins, driving their association with membranes or specific membrane microdomains [1]. S-acylation is reversible by deacylation and can therefore act as a dynamic on/off switch, similar to phosphorylation, acetylation, and ubiquitination. Since the 16-carbon-long palmitic acid is the most frequently added moiety, S-acylation has often been referred to as S-palmitoylation (S-PALM), or simply palmitoylation, even when the specific lipid has not been identified [1]. Over the past two decades, S-PALM has emerged as a crucial regulator of protein function. S-PALM was found to alter the conformation or structure of a protein, intracellular trafficking and membrane localization, protein-protein interactions and signal transduction [2, 3]. This process is mediated by palmitoyl acyltransferase enzymes (PATs) and reversed by thioesterases, (reviewed in [4–6]) and may be potentially important in the context of activity-dependent modification of synapse function. Protein palmitoylation is not only thought to contribute to healthy physiological processes such as learning and memory, but may play a role in neurodegenerative diseases such as Alzheimer’s disease (AD), Parkinson’s disease (PD), Huntington’s disease (HD), amyotrophic lateral sclerosis (ALS) and other neuropsychiatric disorders [4, 7].

In the nervous tissue, S-PALM was extensively studied in major synaptic proteins, such as the scaffolding postsynaptic density protein 95 (PSD95) and gephyrin, as well as ionotropic receptors (α-amino-3-hydroxy-5-methyl-4-isoxazolepropionic acid receptors [AMPARs], N-methyl-D-aspartate receptors [NMDARs], and γ-aminobutyric acid receptors [GABARs]) that mediate the majority of fast excitatory and inhibitory synaptic transmission in the brain [4–6, 8]. Emerging evidence suggests that S-PALM may serve as an additional mechanism to orchestrate synaptic protein distribution, thereby supporting synaptic plasticity, learning and memory [4]. For instance, manipulation of several PATs genes such as ZDHHC3, ZDHHC7, ZDHHC9, or ZDHHC17, was reported to affect basal excitatory and/or inhibitory synaptic transmission [4], while knockout of the gene coding depalmitoylating enzyme PPT1 was shown to affect inhibitory synaptic transmission and presynaptic release [9, 10]. Moreover, knockouts of PATs including ZDHHC2, ZDHHC5, ZDHHC9, and ZDHHC17, as well as PPT1, were linked with impairments in spatial or fear learning [9, 11–13] and pharmacological inhibition of palmitoylation impairs memory acquisition and consolidation in the hippocampus [14]. These findings indicate that palmitoylation may regulate the excitatory-inhibitory balance in neuronal networks and play a vital role in normal brain function, particularly in neuronal plasticity. A recent study revealed a list of > 60 synaptic proteins that could undergo changes in S-PALM 1 h after the formation of a context*-*dependent fear memory in mice [15], suggesting that this process is highly protein-specific. However, the mechanisms underlying protein selectivity in neuronal palmitoylation, the precise temporal dynamics of this modification, and the signals that trigger its regulation remain poorly understood. Unraveling these aspects is critically important, as dysregulated palmitoylation has been implicated in numerous neuropsychiatric conditions.

Functional studies conducted both in vitro and in vivo have confirmed that memory traces can be encoded through activity-dependent modifications of synapses. A defining feature of such synaptic plasticity is long-term potentiation (LTP) or long-term depression (LTD), which are elicited by patterned stimulation of afferent fibers at high and low frequencies, respectively [16]. The most widely studied form of synaptic plasticity is LTP, which leads to long-lasting strengthening of synapses in response to enhanced synapse activity [17] and is considered the biological substrate for learning and memory [18]. Synaptic potentiation can occur through pre- and/or postsynaptic mechanisms and may involve NMDAR subtype of glutamate receptors [19], although some forms of LTP can be expressed independently of NMDAR activity [20, 21]. Regardless of the specific mechanism, most glutamatergic synapses require an initial rise in cytosolic Ca^2+^ concentrations for LTP induction. This increase is mediated via NMDARs, voltage-gated calcium channels (VGCCs), calcium-permeable AMPARs (CP-AMPARs), or release from intracellular calcium stores [17]. The early phase of LTP (E-LTP) primarily depends on protein trafficking to support synaptic potentiation. In contrast, the late phase of LTP (L-LTP) involves longer-lasting, transcription-dependent changes that stabilize synaptic modifications, resulting in sustained synaptic enhancement [22]. Additional factors contribute to LTP induction and maintenance, such as brain-derived neurotrophic factor (BDNF), which facilitates synaptic potentiation by activating the TrkB receptor tyrosine kinase [23].

In this study, we combined electrophysiology, biochemistry, proteomics, and imaging to explore the role of protein palmitoylation in synaptic plasticity and neuronal network activity. We employed various models that are well established in the literature, effective in our hands, and consistently yield reliable results for both plasticity markers and palmitoylation readouts. By inducing plasticity using various chemical and electrical stimulation methods, we mapped the dynamics of palmitoylation, assessed its impact on excitatory synapse function, and identified proteins that are dynamically modified in response to increased neuronal activity. This approach demonstrated that palmitoylation is robustly engaged across all neuronal plasticity models examined. Our findings indicate that palmitoylation supports plasticity of excitatory transmission in the hippocampus in a synapse-specific manner and may influence neuronal spiking and information flow within neural networks. At the molecular level, we observed that synaptic plasticity induction triggers transient and protein-specific changes in palmitoylation.

## Results

### The induction of neuronal plasticity in vitro regulates the palmitoylation of synaptophysin and neurochondrin

**Fig. 1:**
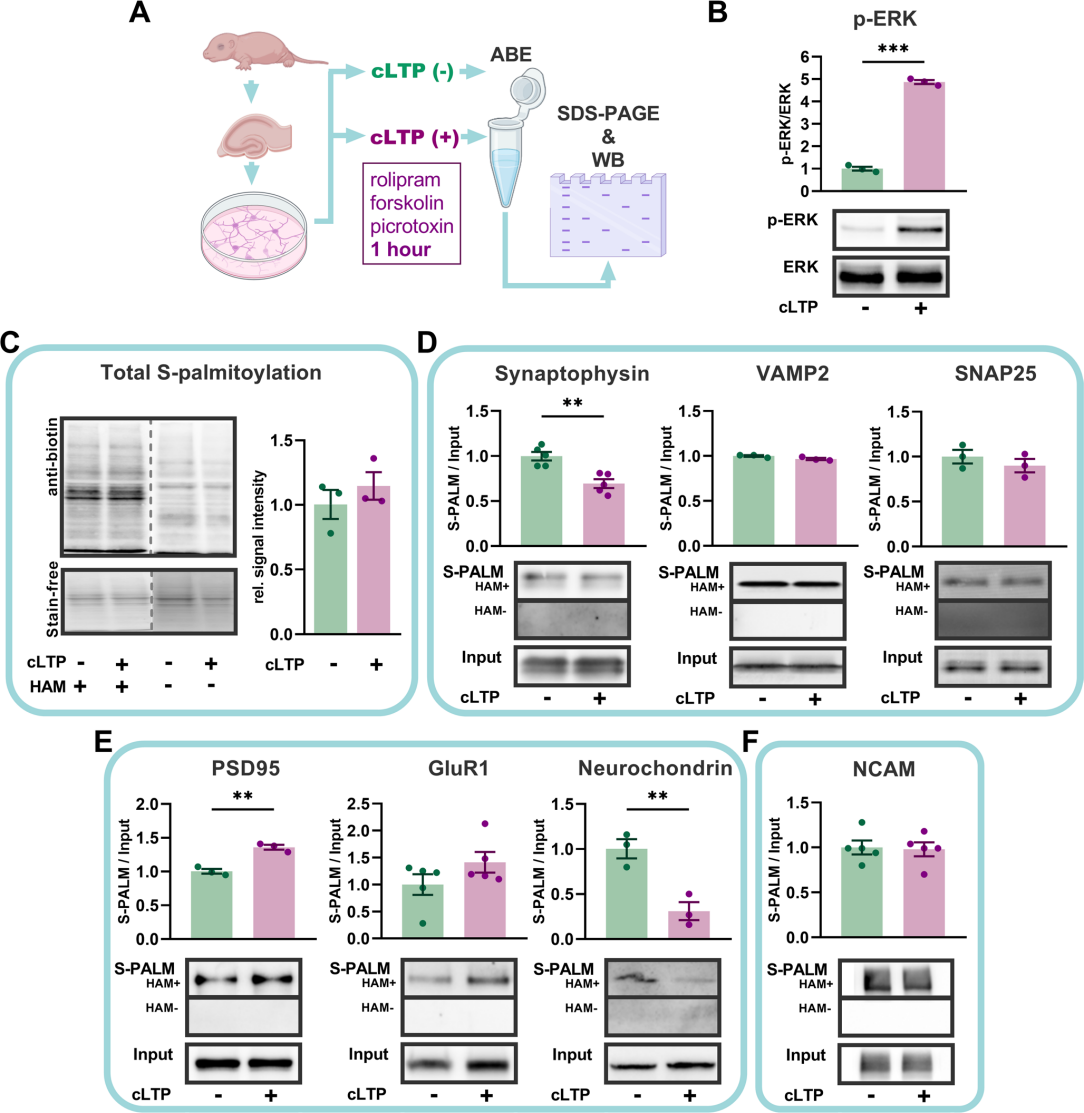
Induction of synaptic plasticity in neuronal cultures regulates palmitoylation of synaptic proteins. **A** Primary rat hippocampal neurons were cultured for 14 days and treated with cLTP cocktail (cLTP +) or solvent (cLTP -). Culture homogenates were collected 1 h after treatment, subsequently subjected to ABE and S-PALM and input fractions were immunoblotted for target proteins, as indicated. **B** Quantification of the phosphorylated extracellular regulated kinase (pERK) protein expression in culture homogenates. cLTP resulted in a significant increase in the expression of pERK protein (*n =* 3 cultures, each culture was prepared from mixed hippocampal neurons of *N* = 12 animals, see Materials and methods section, *p =* 0.002, unpaired Student’s *t-*test). **C** Western blot of global palmitoylation at 1 h post cLTP. HAM stands for hydroxylamine. There was no shift in global protein palmitoylation (*n =* 3 cultures, *p =* 0.40, unpaired Student’s *t-*test). **D**-**E** Western blot and quantification of the ABE assay described in (A) performed on selected pre – and postsynaptic proteins. The levels of palmitoylated synaptophysin and neurochondrin significantly decreased post cLTP (*n =* 5 and 3 cultures respectively, *p =* 0.002 and *p =* 0.009, respectively, unpaired Student’s *t-*test). In contrast, the levels of palmitoylated PSD-95 increased significantly post cLTP (*n =* 3 cultures, *p =* 0.002, unpaired Student’s *t-*test). **F** Western blot and quantification of the ABE assay described in (A) performed on an exemplary bipolar adhesion molecule NCAM. Full images of the blots, including molecular weight markers, are provided in Supplementary Fig. S8. Data are mean ± SEM from *n =* 3 to 5 cultures. Asterisks indicate statistical significance: ** *p <* 0.01, *** *p <* 0.001

Understanding neuronal assemblies is essential for uncovering the mechanisms underlying brain coding, learning, and memory. To explore protein palmitoylation’s role in activity-dependent neuronal spiking and network properties, primary rat hippocampal neurons at 14 days in vitro (DIV) were treated with picrotoxin, forskolin, and rolipram for 1 h to induce long-term synaptic plasticity. This cocktail is known to increase cAMP level and network activity leading to a tetanic-like stimulation in bulk and induction of functional and structural neuronal plasticity [24, 25] (Fig. 1A). This method of neuronal stimulation is referred to as chemically induced long-term potentiation (cLTP). We demonstrated that 1 h after cLTP induction, there is a significant upregulation of phosphorylated extracellular signal-regulated kinase (pERK), a molecular marker of LTP. (Fig. 1B) [26]. We next studied protein palmitoylation and subjected homogenized neuronal cultures to the acyl-biotin exchange (ABE) method [27] and quantified total protein palmitoylation levels as well as that of individual proteins (see methods for details). We observed that cLTP induction did not affect global protein palmitoylation levels (Fig. 1C) when normalized to total protein levels (Stain-free technology). However, it significantly altered the palmitoylation status of specific presynaptic and postsynaptic proteins (Fig. 1D-F). Specifically, we examined three categories of proteins: presynaptic (synaptophysin, vesicle-associated membrane protein 2 [VAMP2], synaptosome-associated protein 25 [SNAP25]), postsynaptic (postsynaptic density protein 95 [PSD95], glutamate ionotropic receptor AMPA type subunit 1 [GluR1], neurochondrin), and inter-synaptic (neural cell adhesion molecule [NCAM]). At 1 h post cLTP induction, the palmitoylation levels of synaptophysin and neurochondrin were significantly decreased (Fig. 1D–F). In contrast, in the same samples, the palmitoylation level of PSD95 was significantly increased, consistent with previous findings [28, 29] while most other proteins we analyzed did not exhibit significant changes (Fig. 1D–F). To further investigate the temporal dynamics of these changes and assess the stability of the palmitoylation profile, we repeated the experiment using a shorter, 20-minute time window, focusing on selected protein representatives from the pre-, inter-, and postsynaptic compartments. Unlike the 1-hour treatment, at 20 min post*-*cLTP induction, the palmitoylation level of synaptophysin increased, that of NCAM decreased, and GluR1 remained unaltered (Fig. S1). Overall, these results demonstrate that the induction of neuronal plasticity via cLTP in primary neuronal cultures leads to time-dependent and protein-specific changes in protein palmitoylation. We indicate synaptophysin, neurochondrin, and NCAM as examples of dynamic regulation by palmitoylation.

### Protein deacylation alters the temporal organization of neuronal spiking following periods of heightened network activity

**Fig. 2:**
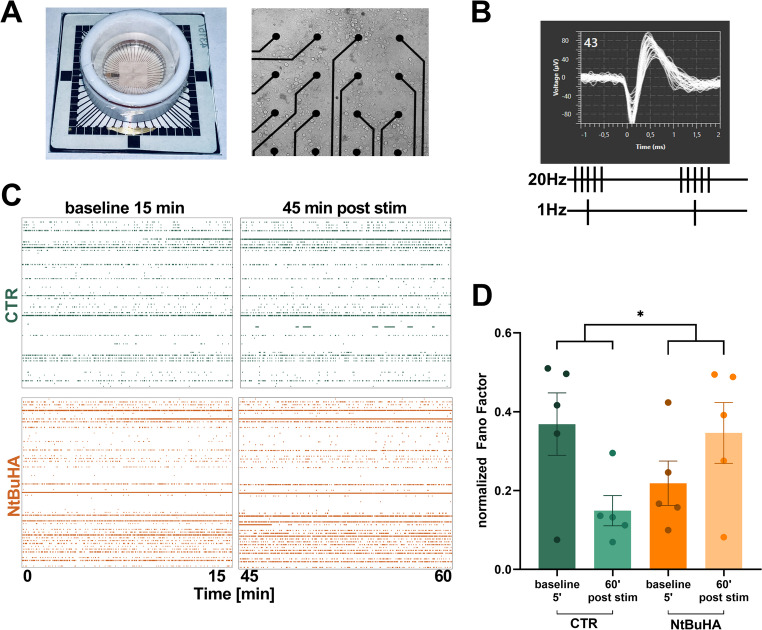
Protein deacylation modulates the temporal organization of neuronal spiking following a period of enhanced network activity. **A** Primary rat hippocampal neurons were cultured for 14 days on multielectrode arrays (MEA), allowing recordings of action potentials in a network of neurons from up to 60 sites (*n* = 5 MEA from 3 separate cultures, each culture was made from mixed neurons of 12 Wistar rat pups). MEAs were covered with fluorinated ethylene-propylene membranes to maintain medium osmolarity and the day before recordings received either vehicle or deacylation agent NtBuHA (1 mM, overnight). **B** Representative waveforms of extracellularly recorded action potentials at 14 DIV together with a scheme of pattern of electrical stimulation. Network plasticity was analyzed up to 30 min before and up to 60 min after electrical stimulation of two non-adjacent electrodes on the MEA (20 Hz bursts paired with 1 Hz in-phase activity for a total of 120 s). **C** Exemplary raster plots show spiking activity over a 15-minute period before and after associative electrical stimulation. Each row corresponds to an electrode, and each dot represents a single action potential. **D** Quantification of spikes recorded in control cultures (green) and cultures treated overnight with NtBuHA (orange) before and after inducing network plasticity through associative activity (stim.). The normalized Fano Factor was analyzed before and after stimulation. A significant interaction between treatment and stimulation was observed (*F*(1,8) = 7.421, *p* = 0.026, two-way ANOVA) but no significant main effects were found. Data are shown as mean ± SEM. *n =* 5 MEAs per group. Asterisk indicates statistical significance (* *p <* 0.05)

We next investigated how protein palmitoylation impacts neuronal spiking in the same model (primary hippocampal cultures). Cells were cultured for 14 days on multielectrode arrays (MEA), allowing for extracellular and non-invasive recordings of action potentials in a network of neurons from up to 60 sites (Fig. 2A-B). These multisite recordings facilitate the capture of spiking changes at the population level while allowing for the manipulation of neuronal activity via electrical stimulation (description of basic parameters of MEA recordings are shown in Fig. S2).

To induce neuronal plasticity in MEA, we employed a previously established electrical stimulation protocol [30]. This paradigm was selected for two primary reasons. First, in this approach, neurons remain in the incubator and are recorded without direct intervention by the experimenter. Second, our preliminary attempts to induce network plasticity using chemically induced long-term potentiation (cLTP, Fig. 1) proved unreliable, yielding inconsistent results of either potentiation or depression of neuronal spiking (data not shown). Perhaps it was partly because the cLTP protocol required removing the microelectrode array (MEA) from the incubator, exchanging the medium, and mixing, which could influence neuronal spiking within a short time frame.

To manipulate protein palmitoylation, at 13 DIV we treated neurons overnight with deacylation agent N-(tert*-*Butyl)hydroxylamine (NtBuHA), a hydroxylamine derivative [31]. As shown in Fig. S3A, the chemiluminescent signal corresponding to total proteome palmitoylation in culture homogenates was significantly reduced following an overnight treatment with NtBuHA (1 mM). Moreover, NtBuHA at 1 mM, as well as 2-bromopalmitate (2-BP) (100 µM) - an irreversible and nonselective inhibitor of palmitoyl acyltransferases [4, 32], did not induce cellular toxicity, as indicated by the absence of increased lactate dehydrogenase (LDH) release (Fig. S3B). To further evaluate the efficacy of NtBuHA as a deacylation agent, total proteome palmitoylation in neuronal cultures was assessed using a click chemistry reaction with Oregon Green 488 dye [33]. Primary rat hippocampal cultures at 13 DIV were treated overnight with exogenous alkyne-palmitic acid to facilitate its incorporation into cellular proteins. Some cultures were additionally exposed to 1 mM NtBuHA. As shown in Fig. S3C-D, NtBuHA significantly reduced fluorescence associated with labeled exogenous palmitate to the level of negative control (cultures treated only with the fluorophore), further confirming its effectiveness in globally deacylating neuronal proteins. Based on these findings, we utilized water-soluble NtBuHA (1 mM) in subsequent experiments.

Consequently, we applied paired associative activity at two electrodes within the network of hippocampal neurons for 2 min (AT-IN protocol, Fig. 2C, see Methods [30]) and subsequently monitored network activity for 1 h. Such a period of enhanced neuronal activity or the presence of NtBuHA did not affect the number of active electrodes, number of bursts or the average spike frequency (Fig. S2). However, electrical stimulation had a significant impact on the temporal organization of spiking (Fig. 2C-D). The Fano Factor reflects the variability in spike occurrence relative to the average number of spikes. In the CTR group, the Fano Factor significantly decreased over time suggesting that the network became more organized with more regular or rhythmic firing. In contrast, following deacylation with NtBuHA, an inverse effect was observed (Fig. 2D). Altogether, protein deacylation had no effect on the mean rate or temporal distribution of action potentials in the neuronal network, but it impaired changes in the temporal organization of neuronal spiking following a period of enhanced network activity.

## NMDAR-dependent synaptic potentiation in the hippocampus results in rapid and protein-specific palmitoylation

**Fig. 3:**
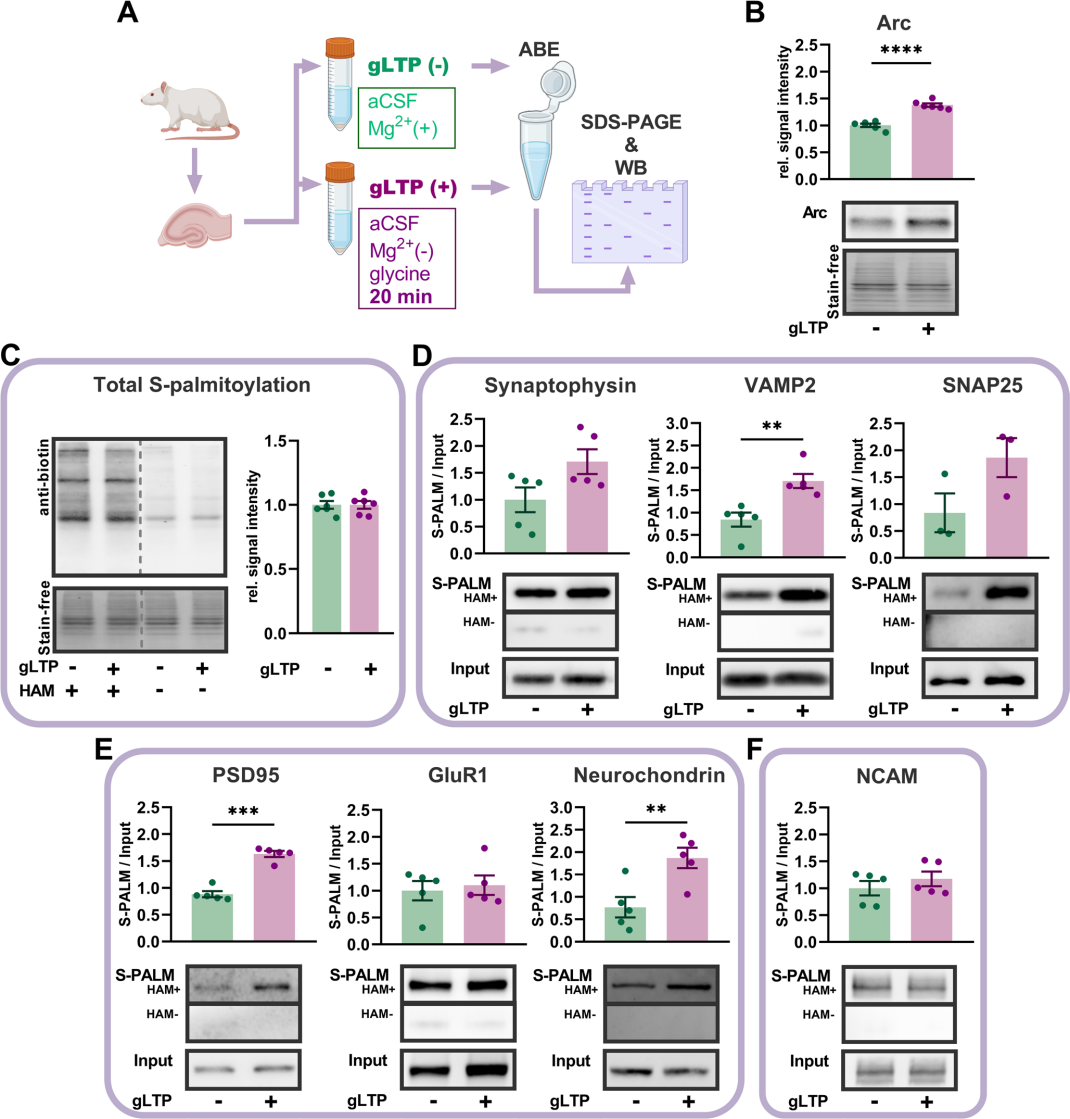
Glycine-induced LTP (gLTP) in hippocampal slices induces the differential palmitoylation of proteins. **A** Schematic illustration of the NMDAR-dependent, glycine-induced LTP assay (gLTP) in hippocampal slices to investigate activity-induced changes in neuronal protein palmitoylation. Tissue homogenates were collected 20 min after treatment with either mock gLTP (−) or gLTP (+). Both S-PALM and input fractions were immunoblotted for target proteins, as indicated. **B** Quantification of the Arc protein expression in tissue homogenates. gLTP resulted in a significant increase in the expression of Arc protein (*n =* 6 animals, ***p <*** **0.0001**, unpaired Student’s *t-*test). **C** Western blot of global palmitoylation at 20 min post gLTP. HAM stands for hydroxylamine. gLTP did not affect global protein palmitoylation levels (*n =* 6 animals, *p =* 0.99, unpaired Student’s *t-*test). **D** Western blot and quantification of the ABE assay described in (A) performed on selected presynaptic proteins. The levels of palmitoylated VAMP2 increased significantly post cLTP (*n =* 5 animals, *p =* 0.004, unpaired Student’s *t-*test). **E** Western blot and quantification of the ABE assay described in (A) performed on selected postsynaptic proteins. The levels of palmitoylated PSD-95 and neurochondrin increased significantly post cLTP (*n =* 5 animals, *p <* 0.0001 and *p =* 0.009, respectively, unpaired Student’s *t-*test). **F** Western blot and quantification of the ABE assay described in (A) performed on an exemplary bipolar adhesion molecule NCAM. Full images of the blots, including molecular weight markers, are provided in Supplementary Fig. S8. Data are mean ± SEM. Asterisks indicate statistical significance: ** *p <* 0.01, *** *p <* 0.001, **** *p <* 0.0001

Next, we analyzed protein palmitoylation in acute hippocampal slices following LTP induction to obtain functional validation in a near-native context. This model preserves in vivo-like architecture and connectivity, provides a more physiological extracellular environment, and maintains intact glia-neuron interactions and molecular signaling pathways, including PTMs, compared to dissociated neuronal cultures. To induce NMDAR-dependent LTP, we incubated hippocampal slices in Mg^2+^-free aCSF with addition of glycine (600 µM) for 10 min and subsequently maintained the slices in the same aCSF without glycine for 20 min (glycine LTP, gLTP) [34]. To confirm the efficacy of this protocol, we performed recordings of evoked field excitatory postsynaptic potentials (fEPSPs) in response to Shaffer collaterals stimulation. We recently discovered that the molecular mechanisms of synaptic plasticity and the protein involvement in neighboring excitatory connections on basal and apical dendrites in CA1 hippocampal region differ significantly [35], similarly to synapses in pyramidal neurons of CA3 region [20]. Therefore, we looked at synaptic signals in *stratum oriens* (SO) and *stratum radiatum* (SR) (hubs to excitatory synapses located in basal or apical dendrites of pyramidal neurons, respectively). As shown in the Fig. S4, the application of glycine at time 0 for 10 min resulted in significant potentiation of fEPSP amplitudes in both regions. To confirm whether gLTP is NMDAR-dependent, we repeated recordings in SR with the addition of the NMDAR competitive antagonist APV (50 µM) to the glycine solution. In the presence of APV, the potentiating effect of glycine was not observed (Fig. S4). In addition, fEPSP amplitudes recorded in response to a wide range of stimuli were significantly increased in the presence of glycine in both SR and SO but not when glycine was co-applied with APV (Fig. S4B-E). We also found in hippocampal slice homogenates that gLTP resulted in a significant upregulation of activity-regulated cytoskeleton-associated protein (Arc), a molecular marker of LTP (Fig. 3B) [36]. Altogether, these results confirm that glycine-induced synaptic plasticity in acute hippocampal slices occurs in an NMDAR-dependent way and provide a reliable model to study protein-specific palmitoylation following synaptic potentiation.

We next investigated how gLTP affected palmitoylation of synaptic proteins in slice homogenates subjected to the ABE method [27] (see methods for details). We found that gLTP did not affect global protein palmitoylation chemiluminescent signal (Fig. 3C) when normalized to the total protein signal (Stain-free technology). However, it significantly upregulated the palmitoylation level of certain presynaptic or postsynaptic proteins. In particular, VAMP2, neurochondrin and PSD-95 palmitoylation levels were significantly increased (Fig. 3D-E). Palmitoylation levels of synaptophysin and SNAP25 showed an upward trend, although these changes did not reach statistical significance. Altogether, these results demonstrate that NMDAR-dependent synaptic plasticity in hippocampal slices induces rapid and protein-specific upregulation of palmitoylation, highlighting the dynamic regulation of lipid modifications in synaptic function.

### Protein depalmitoylation differentially affects long-term synaptic potentiation in hippocampal excitatory synapses

**Fig. 4:**
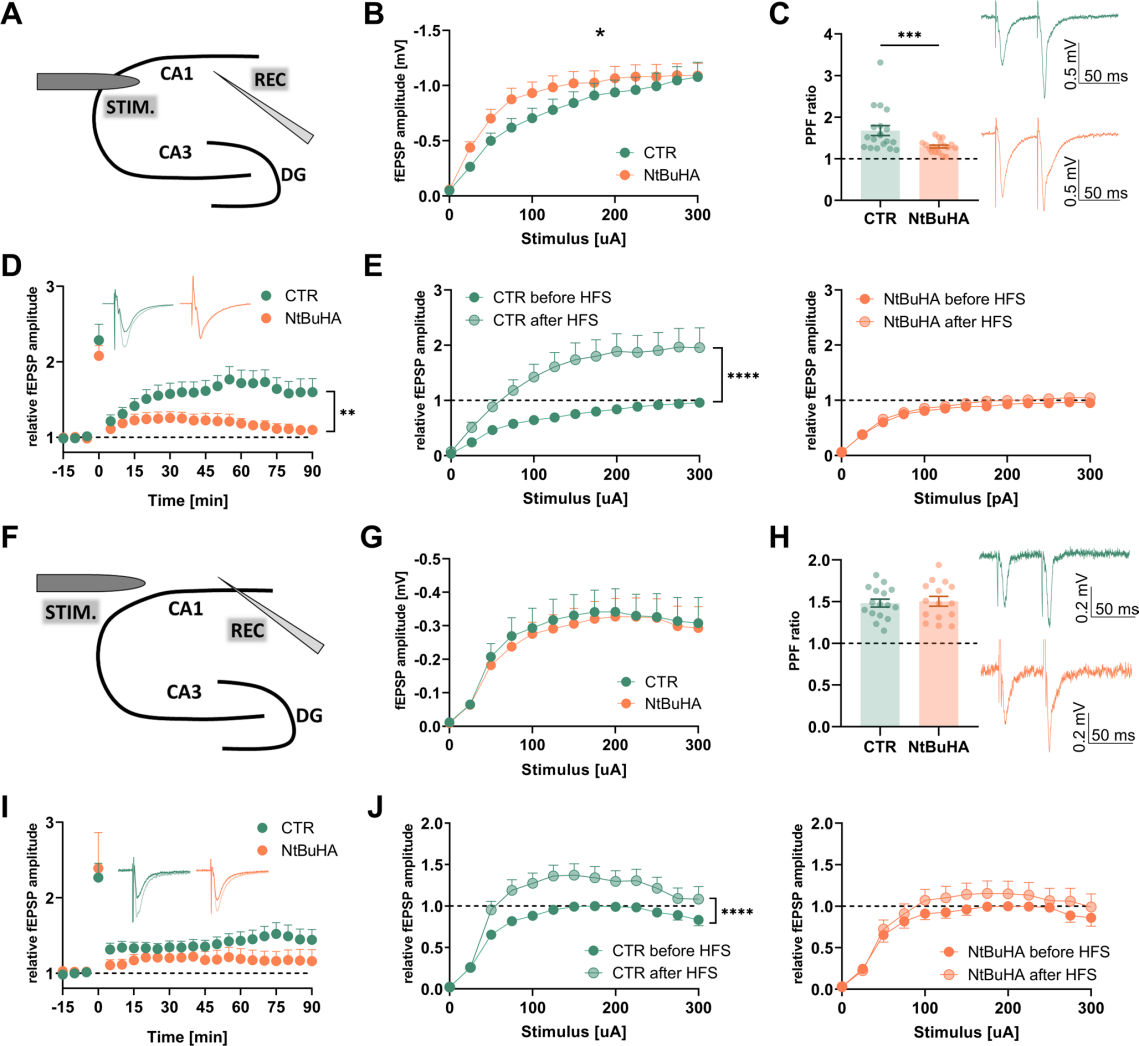
Protein depalmitoylation differentially affects synaptic plasticity in hippocampal excitatory synapses. **A** Schematic representation of fEPSP recordings in SR excitatory synapses in response to Schaeffer collaterals stimulation (A-E). **B** Statistical analysis of fEPSP amplitudes recorded in response to extracellular stimulation in the control slices and those incubated with depalmitoylating agent NtBuHA. Note that in the presence of the drug, fEPSP amplitudes were increased (*n =* at least 17 slices per group, *F*_(12, 396)_ = 2.044, *p =* 0.019, two-way ANOVA, treatment × stimulus intensity). **C** Statistical evaluation of fEPSP recordings in response to paired-pulse stimulation (50 ms inter-stimulus-interval). It is noteworthy that in the presence of the drug, the index is significantly decreased (*p =* 0.0052, unpaired-*t-*test, *n* = 19/15 slices, *N* = 15/14 animals in control/NtBuHA groups, respectively). Insets show exemplary recordings with matching colors. **D** Exemplary traces of fEPSPs and the time course of fEPSPs recorded before and after LTP induction with high frequency stimulation (hfsLTP, 4 × 100 Hz, applied at time = 0 min). In the presence of NtBuHA, the magnitude of hfsLTP at 90 min was significantly decreased (*n =* 15/16 slices, *N* = 15/13 animals for CTR and NtBuHA groups, respectively, *p =* 0.013, unpaired *t-*test). **E** Statistics of fEPSP responses to monotonically increased stimuli before (full color) and 90 min after hfsLTP induction (transparent color). In control slices, hfsLTP resulted in significant upregulation of fEPSP amplitudes in response to a wide range of stimuli (left panel, *F*_(12, 408)_ = 6.449, *p <* 0.0001, two-way ANOVA, treatment × stimulus intensity) and this was not observed in slices treated with NtBuHA (right panel, *F*_(12, 384)_ = 1.005, *p =* 0.44, two-way ANOVA). **F** Schematic presentation of fEPSP recordings in SO excitatory synapses in response to Schaeffer collaterals stimulation (G-J). **G** Statistical analysis of fEPSP amplitudes recorded in response to extracellular stimulation. There was no difference between the control slices and those incubated with the depalmitoylating agent NtBuHA (*n =* 15/16 slices, *N* = 15/16 animals per CTR and NtBuHA group, respectively, *F*_(12, 348)_ = 0.07, *p* > 0.99, two-way ANOVA, treatment × stimulus intensity). **H** Statistics of fEPSP recordings in response to paired-pulse stimulation (50 ms inter-stimulus-interval) indicating no difference between investigated groups (*p =* 0.78, unpaired-*t-*test). Insets show exemplary recordings with matching colours (*n =* 15/16 slices, *N* = 15/16 animals per CTR and NtBuHA group, respectively). **I** Exemplary traces of fEPSPs and the time course of fEPSPs recorded before and after hfsLTP induction with high-frequency stimulation (4 × 100 Hz, applied at time = 0 min). In the presence of NtBuHA, the magnitude of hfsLTP at 90 min was not significantly different from controls (*n =* 15/16 slices, *N* = 15/16 animals per CTR and NtBuHA group, respectively, *p =* 0.15, unpaired *t-*test). **J** Statistics of fEPSP responses to monotonically increased stimuli before (full color) and 90 min after hfsLTP induction (transparent color). In control slices, hfsLTP resulted in significant upregulation of fEPSP amplitudes in response to a wide range of stimuli (left panel, *F*_(12, 360)_ = 2.854, *p =* 0.0009, two-way ANOVA, treatment × stimulus intensity), which was not observed in slices treated with NtBuHA (right panel, *F*_(12, 336)_ = 0.417, *p =* 0.95, two-way ANOVA, treatment × stimulus intensity). Asterisks indicate statistical significance: * *p <* 0.05, ** *p <* 0.01, *** *p <* 0.01, **** *p <* 0.0001

Next, we studied whether involvement of protein palmitoylation in synaptic plasticity depends on the type of excitatory synapse. Before electrophysiological recordings, slices were bathed in NtBuHA (1 mM) for 2 h. Incubation of hippocampal slices with NtBuHA significantly downregulated protein palmitoylation in homogenates, as shown by the ABE method (Fig. S5A-B). fEPSPs were then evoked in SO or SR in response to Schaffer collaterals stimulation (Fig. 4A, F; see Materials and methods for details). As shown in Fig. 4B, G, fEPSPs amplitudes recorded in response to monotonically increasing stimuli (input*-*output curves) were significantly increased in the presence of NtBuHA in SR but not in SO. Next, we studied short*-*term synaptic plasticity by stimulating afferents with two pulses (inter-event interval of 50 ms). We observed a significant reduction of paired-pulse facilitation ratio in SR in the presence of NtBuHA but not in SO (Fig. 4C, H). Altogether, in the presence of the deacylating agent NtBuHA, basal synaptic transmission and short*-*term facilitation were affected preferentially in SR but not in SO.

To study the impact of protein deacylation on long-term synaptic plasticity we applied high-frequency stimulation (4 × 100 Hz) of Schaffer collaterals (hfsLTP). We found that in control (CTR) slices, the relative fEPSP amplitude 90 min post hfsLTP induction was significantly impaired in the presence of NtBuHA in SR but not in SO (Fig. 4D, I). Induction of hfsLTP resulted in a significant leftward shift in the input*-*output function in control slices and this shift was not observed in the presence of NtBuHA both in SR and SO (Fig. 4E, J). In summary, protein depalmitoylation affected the basal transmission, time-course of LTP and short-term synaptic plasticity in SR but not in SO.

### Excitatory synapses contain palmitoylation machinery responsive to external stimuli

**Fig. 5:**
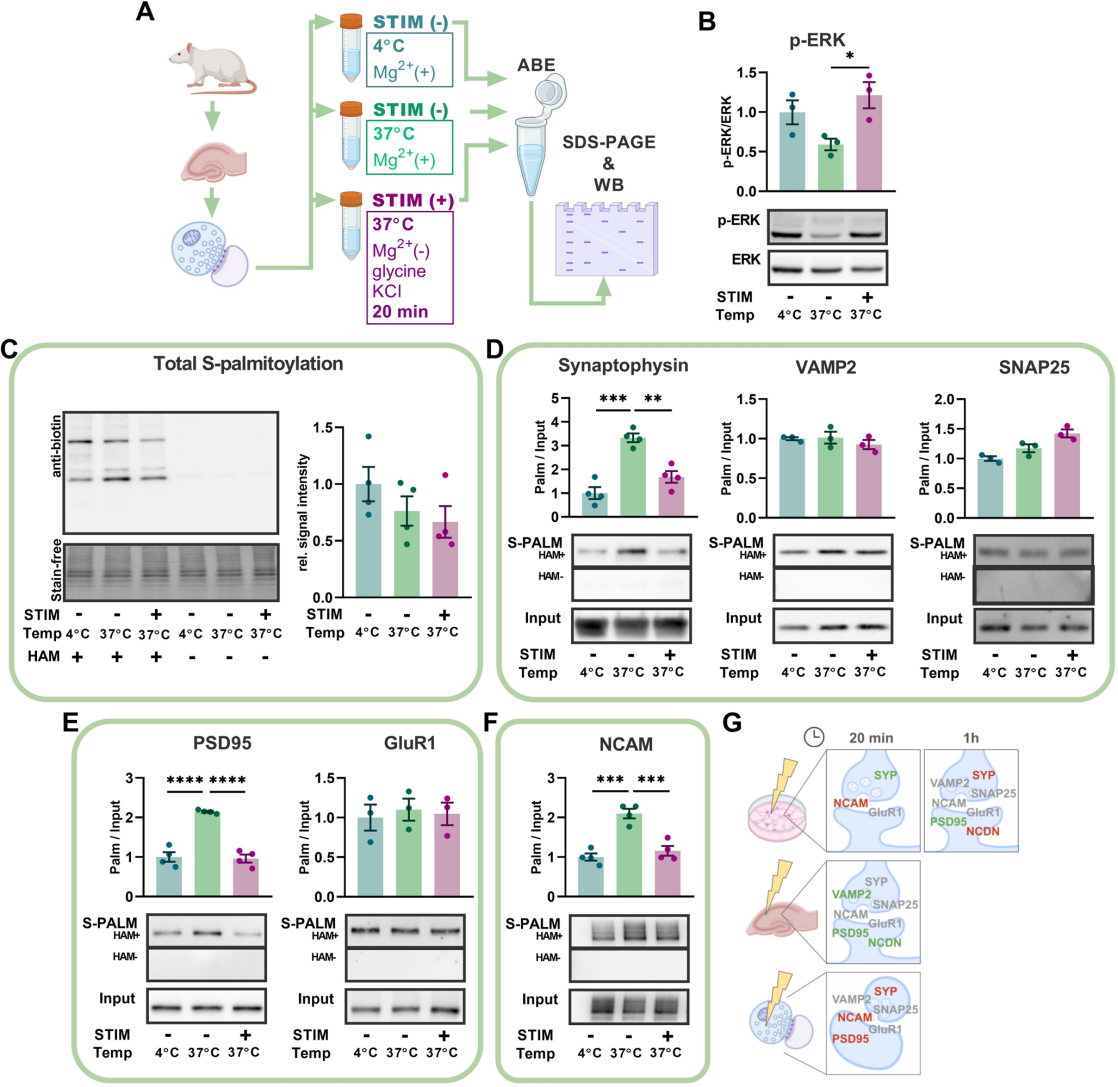
Synaptoneurosomes contain palmitoylation machinery responsive to external stimuli. **A** Schematic illustration of the NMDAR-dependent glycine and KCl-induced stimulation assay (STIM) in live synaptoneurosomes from hippocampi of young rats to investigate activity-induced changes in synaptic protein palmitoylation (for each sample, homogenates from hippocampi of 2 rats were made). Synaptoneurosomes were either kept on ice (4 °C) or treated with either sham (−) or STIM (+) for 20 min. Both S-PALM and input fractions were immunoblotted for target proteins, as indicated. **B** Quantification of the pERK/ERK protein expression in synaptoneurosomes. STIM resulted in an increase in the level of phosphorylated ERK compared to mock solution (-) (*n =* 3 samples, *N* = 6 animals, *F*_(2, 6)_ = 5.42, *p =* 0.045, one-way ANOVA). **C** Western blot of global palmitoylation indicates no global change in protein palmitoylation of synaptoneurosomes post STIM (*n =* 4 samples, *N* = 8 animals, *F*_(2, 9)_ = 1.486, *p =* 0.27, one-way ANOVA). **D** Western blot and quantification of the ABE assay described in (A) performed on selected presynaptic proteins. The levels of palmitoylated synaptophysin decreased significantly post STIM (*n =* 4 samples, *N* = 8 animals, *F*_(2, 9)_ = 27.49, *p* < 0.001, one-way ANOVA). **E** Western blot and quantification of the ABE assay described in (A) performed on selected postsynaptic proteins. The level of palmitoylated PSD95 decreased significantly post STIM (*n =* 4 samples, *N* = 8 animals, *F*_(2, 9)_ = 53.31, *p <* 0.001, one-way ANOVA). **F** Western blot and quantification of the ABE assay described in (A) performed on exemplary bipolar adhesion molecule NCAM. The level of palmitoylated NCAM decreased significantly post STIM (*n* = 4 samples, *N* = 8 animals, *F*_(2, 9)_ = 27.79, *p <* 0.001, one-way ANOVA). **G** Graphical summary of results obtained in experimental models of synaptic plasticity: neuronal cultures, hippocampal slices and synaptoneurosomes. The change in palmitoylation levels relative to the control for each protein is indicated in red (decrease), green (increase) or gray (no change). Full images of the blots, including molecular weight markers, are provided in Supplementary Fig. S8. Data are mean ± SEM. Asterisks indicate statistical significance: * p < 0.05, ** *p <* 0.01, *** *p <* 0.001, **** *p <* 0.0001

Most PATs are transmembrane enzymes located in the Golgi apparatus and the endoplasmic reticulum [4]. In dendrites and spines several members of the PATs family such as ZDHHC2, ZDHHC5, ZDHHC8, and ZDHHC17 have been suggested to regulate the location and function of multiple signaling proteins [6, 29]. Given the presence of PATs in dendrites and spines, we investigated whether isolated synaptoneurosomes contain active palmitoylation machinery capable of responding dynamically to external stimuli. Synaptoneurosomes contain both presynaptic (synaptosome) and postsynaptic (neurosome) vesicularized components, and are widely used to study synaptic structure and function [37]. It was reported that isolated synaptoneurosomes are functional and can undergo LTP-like plasticity in response to stimuli that mimic synaptic NMDAR activation [38]. In this protocol (see scheme, Fig. 5A), KCl-evoked release of endogenous glutamate from presynaptic terminals, in the presence of the NMDAR co-agonist glycine, leads to a long-lasting increase in surface AMPAR levels, as shown previously [38].

We took advantage of this model to study synapse-restricted protein palmitoylation. To this end, synaptoneurosomes from rat hippocampi were prepared in ice-cold solutions (for each sample, homogenates from hippocampi of 2 rats were used, see Materials and methods, Fig. 5A). Our preparations showed 3–4 fold enrichment in PSD95 compared to gephyrin (Fig. S6). This extends our previously reported 1.5- to 4-fold enrichment in excitatory synaptic proteins, including PSD95, GluA1, GluA2, and Neuroligin 3 [39]. Synaptoneurosomes were warmed at 37 °C for 10 min, and subsequently treated with a stimulating solution (STIM) containing glycine and 20 mM KCl for 20 min [38] (see Materials and methods for details). Additionally, for each replicate part of the material was left on ice (4 °C) throughout the experiment to check the effect of temperature on protein stability and palmitoylation. The STIM protocol resulted in a significant upregulation of the level of phosphorylated extracellular signal regulated kinase (pERK), a molecular marker of LTP (Fig. 5B) when compared to the control sample 37 °C [26]. Having confirmed that synaptoneurosomes are viable and responsive to extracellular stimuli (temperature and STIM), we used the ABE method on synaptoneurosomal fractions to analyze palmitoylation levels of proteins. We found that stimulation did not affect global protein palmitoylation in synaptoneurosomes (Fig. 5C). However, we found that in contrast to neuronal cultures and slices, most investigated proteins exhibited depalmitoylation following stimulation. In particular, synaptophysin, PSD95 and NCAM palmitoylation levels were significantly decreased (Fig. 5D-F). Altogether, the stimulation of synaptoneurosomes with glycine and KCl led to rapid and protein-specific downregulation of palmitoylation. Additionally, a graphical summary of the changes in palmitoylation for all the proteins investigated in above mentioned models (cultures, slices and synaptoneurosomes) is shown in Fig. 5G.

**Fig. 6:**
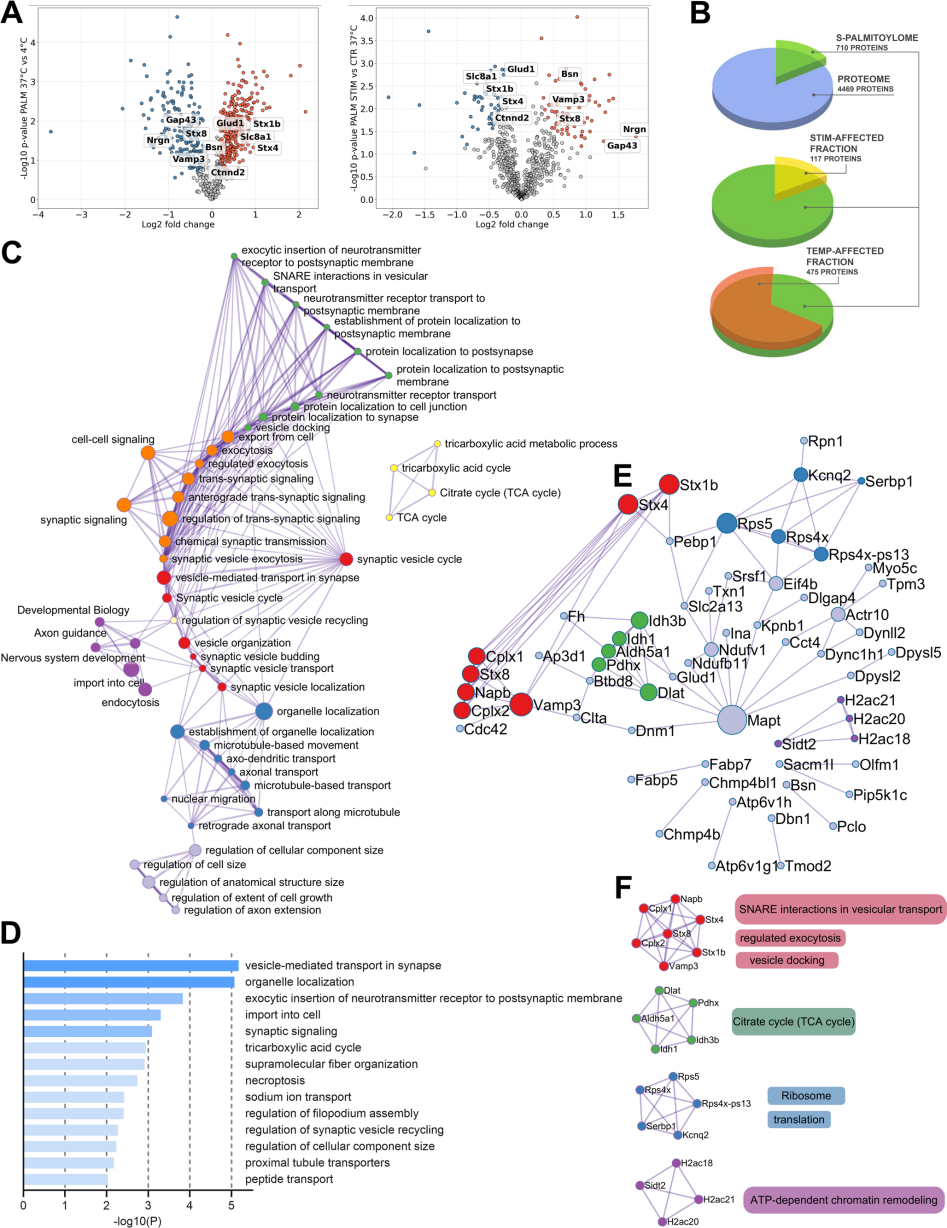
Mass spectrometry-based characterization of protein palmitoylation levels in rat hippocampal synaptoneurosomes following a 20-min stimulation (STIM) with KCl and glycine (protein names have been converted to corresponding gene names throughout the figure for clarity and ease of presentation). **A** Left panel: Volcano plot comparing control samples incubated at 4 °C (CTR 4 °C) versus 37 °C (CTR 37 °C) in Mg²⁺-containing aCSF (*n* = 4 samples per group; each sample was prepared from hippocampal homogenates pooled from two rats). A paired, two-sided Student’s t-test (S₀ = 0.1, FDR = 0.05) was used to identify proteins with significant changes in palmitoylation. The X-axis represents fold change in palmitoylation; the Y-axis shows the -log₁₀ (p-value). Red and blue dots indicate proteins with significantly increased and decreased palmitoylation, respectively. Right panel: Volcano plot showing changes in palmitoylation levels in hippocampal synaptoneurosomes following stimulation with KCl (50 mM) and glycine (100 µM) in Mg²⁺-free solution (PALM STIM 37 °C group) compared to control samples incubated in the presence of Mg²⁺ without stimulation (CTR 37 °C). **B** Quantitative summary of the data shown in panel (A). (C-F) Pathway and process enrichment and protein-protein interaction enrichment analyses of differentially palmitoylated proteins (PALM STIM 37 °C versus CTR 37 °C) performed using the Metascape [40] portal and visualized with Cytoscape software [41]. A total of 117 genes encoding palmitoyl proteins were analyzed with 4,502 genes encoding proteins detected in the input samples serving as a background for enrichment calculations. **C** Graphical representation of the enriched terms. Each term is represented by a node, where its size is proportional to the number of genes that fall under that term, and its color represents its cluster identity (i.e., nodes of the same color belong to the same cluster). Terms with a similarity score > 0.3 are linked by an edge (the thickness of the edge represents the similarity score). The network is visualized with Cytoscape with radial layout. **D** Graph of the top statistically significant terms within clusters presented hierarchically based on Kappa-statistical similarities among their gene memberships. **E** Protein-protein interaction (PPI) enrichment analysis performed with Metascape. Only experimentally validated physical interactions from STRING (physical score > 0.132) and BioGRID were included. The resulting network shows proteins that form physical interactions with at least one other protein in the list. **F** For networks containing between 3 and 500 proteins, the Molecular Complex Detection (MCODE) algorithm was applied to identify densely connected subnetworks (neighborhoods where proteins are densely connected). See the Metascape [40] documentation for methodological details

To further investigate the palmitoylome of synaptoneurosomes, we performed a mass spectrometry-based proteomic analysis of the same samples (for each sample, homogenates from hippocampi of 2 rats were used, see Materials and methods for details; Data S1). This analysis of the baseline proteome identified 4,469 proteins, including two palmitoyltransferases (ZDHHC17 and ZDHHC5) and six thioesterases (e.g., Lypla1, Lypla2, Ppt1, and Abhd17a-c) (Data S1). Protein profiles detected by mass spectrometry were highly similar within groups, as evidenced by the clear clustering observed in principal component analysis (PCA), reflecting reproducible and condition-specific palmitoylation patterns (Fig. S7A). The expression level of 90 proteins (2%) was altered following STIM and they were mainly ribosomal proteins. All the palmitoyl-proteins were normalized to their input. Among the 4,469 proteins detected, 710 were palmitoylated, accounting for 15.8% of the identified proteome (see Data S1, Fig. 6B). Notably, 117 proteins were differentially palmitoylated in STIM 37 °C compared to control 37 °C (16.4%, Fig. 6A-B). In part (34 of 117, 29%), these were proteins that changed their palmitoylation status in response to membrane depolarization by KCl and were not altered by varying temperature conditions (4 °C vs. 37 °C). Interestingly, among the 117 differentially palmitoylated proteins in STIM samples compared to controls, 42.7% exhibited depalmitoylation and 57.3% showed increased palmitoylation. This indicates that the response to a single stimulus (KCl-mediated membrane depolarization) can lead to divergent palmitoylation changes at the level of individual proteins within excitatory synapses. The list of exemplary differentially palmitoylated proteins implied in synaptic plasticity included synaptic vesicle fusion and neurotransmitter release proteins (complexins, dynamin-1, VAMP3, bassoon, piccolo), active cytoskeleton remodeling proteins (cofilin, drebrin, GAP43), cytoskeleton dynamics regulators (cofilin-1, complexins, drebrin, dynamin-1, DLGAP4), and proteins involved in calcium signaling (neurocalcin and neurogranin). In addition, we identified neuronal cell adhesion molecule NrCAM and neurotrimin which are known to interact between pre- and postsynaptic sites (Data S1). We next used the Metascape portal, which integrates over 40 independent knowledge bases and provides comprehensive functional enrichment and interactome analysis of proteomic data [40]. Our analysis revealed that clusters of functionally connected and differentially palmitoylated proteins were predominantly associated with synaptic vesicle release and signaling, mitochondrial function, and protein translation (Fig. 6C–E). Interestingly, the largest and most interconnected cluster included presynaptic proteins such as syntaxins, complexins, and VAMP3. Protein-protein interaction enrichment analysis and application of molecular complex detection algorithms within Metascape indicated a low number of highly interacting proteins, which again formed clusters related to presynaptic release, mitochondrial respiration and protein translation (Fig. 6E-F). In agreement with this, STRING analysis of the 117 differentially palmitoylated proteins indicated that most belonged to diverse families with limited physical interaction. An exception was the aforementioned complex cluster of presynaptic proteins (Fig. S7).

Comparison of results obtained using ABE coupled to mass spectrometry (ABE-MS, Fig. 6) with ABE followed by Western blot (ABE-WB, Fig. 5) confirmed palmitoylation of many proteins, including synaptophysin, NCAM, neurochondrin, GluR1, PSD95, SNAP25, and VAMP2. However, the ABE-MS and ABE-WB methods differed in quantitative results. In particular, ABE-MS methodology revealed modest, statistically non-significant reductions (up to 23%, Data S1) in the palmitoylation of synaptophysin and PSD95 following stimulation with KCl/glycine, while the ABE-WB approach showed robust decreases (up to 120%, Fig. 5) in the palmitoylation of these proteins under the same conditions. In summary, our findings demonstrate that excitatory synapses may house key enzymes of the palmitoylation machinery that respond to extracellular stimuli. Specifically, stimulation with glycine and KCl led to the protein-specific addition or removal of palmitate.

## Discussion

In this study, we provide evidence that protein palmitoylation regulates functional short*-*and long-term synaptic plasticity as well as neuronal spiking of neural networks following enhanced neuron activity. We show that following induction of synaptic plasticity, palmitoylation of synaptic proteins undergoes dynamic regulation in protein-specific and time-dependent manner. The distinct roles of palmitoylation in SR versus SO synapses highlight the complexity of synaptic plasticity regulation, suggesting that different signaling pathways are engaged depending on the dendritic compartment. Finally, we provide evidence that isolated excitatory synapses contain active palmitoylation machinery responsive to external stimuli.

### Protein-specific palmitoylation supports synaptic plasticity

It is well-established that protein palmitoylation in neurons is critically dependent on neuronal activity [4]. However, global analysis of palmitoylome status of the nervous system following enhanced neuronal activity is scarce. In one recent study, stimulation of acute hippocampal slices inducing NMDAR-dependent LTP was reported to markedly increase global protein palmitoylation 30–90 min post LTP induction [42]. This would suggest that palmitoylation is a unidirectional process. In contrast to this study, we do not observe a global shift in total palmitate-related signal in neuronal culture or slice homogenates following their pharmacological stimulation. In line with this, our mass spectrometry data support this finding for a broad range of proteins (Fig. 6). Similarly, samples of mice hippocampi analyzed 1 h post learning in a fear conditioning (aversive learning) paradigm showed that only 35 out of 345 proteins were affected (ca. 10%) [15]. Also, the number and direction of palmitoylation for individual proteins in neurons following glutamate treatment or kainic acid-induced seizures were selective and limited to specific proteins [28]. Thus, protein palmitoylation following enhanced neuronal activity appears to remain a rather subtle and target*-*specific process.

In this study with ABE-WB approach we found that presynaptic, postsynaptic or inter-synaptic proteins undergo time- and protein-dependent S-PALM in three different experimental models. With regard to presynaptic proteins, we found that unlike VAMP2 or SNAP25, synaptophysin is dynamically palmitoylated upon enhanced neuronal activity in cultured neurons, hippocampal slices and synaptoneurosomes. Synaptophysin is known for regulating neurotransmitter release and implicated in both exocytosis and endocytosis [43, 44] and its expression in adult rat hippocampus seems to be similar in SR and SO [45]. Interestingly, synaptophysin is involved in the clathrin-dependent endocytosis of synaptic vesicles and is required to sustain fusion events during repetitive stimulation [46]. It also interacts with synaptobrevin and can regulate the fusion of the SNARE complex assembly through sequestration of the VAMP proteins [47]. Additionally, synaptophysin undergoes phosphorylation upon LTP which enhances glutamate release and supports KCl-induced LTP [48]. Synaptophysin requires mobility and relocation away from the active zone and toward the perisynaptic region where endocytosis takes place during LTP [49]. Thus, we speculate that depalmitoylation of synaptophysin could help increase mobility of this protein between compartments and interfering with this process adversely affects short*-*term presynaptic neurotransmitter release. In support of this, we also observed that protein depalmitoylation with NtBuHA resulted in reduced short*-*term synaptic plasticity in SR (Fig. 4). Since paired-pulse facilitation predominantly affects presynaptic release machinery [50] this supports the view that protein palmitoylation may have an important impact on presynaptic release in SR. Indeed, blocking depalmitoylating enzymes or knocking out the APT-1 gene were reported to result in increased frequency of mEPSC indicative of increased presynaptic probability of release [51]. Moreover, PPT1 knockout caused a decline in the number of readily releasable synaptic vesicles [10]. Thus, a picture emerges, whereby palmitoylation may support short*-*term synaptic plasticity in a synapse-dependent manner. On the other hand, synaptophysin contains four transmembrane domains and due to its high abundance in the presynaptic compartment it is often used as presynaptic terminal marker. Therefore over a short time period, palmitoylation could rather modify the function of synaptophysin and not its localization, yet the possible mechanism remains elusive.

Here, we found that palmitoylation of neurochondrin is affected by induction of neuronal plasticity in culture and slices. Neurochondrin (also known as norbin, encoded by the NCDN gene) is expressed specifically in neurons and present in somato-dendritic compartment [52]. Present in the cytosol, it regulates synaptic plasticity, neuronal morphology, and signaling pathways essential for learning and memory (reviewed in [53]). Neurochondrin in dendritic spines associates with actin rather than postsynaptic density proteins such as PSD-95, which suggests a structural role in synaptic function [54]. Indeed, intracellularly neurochondrin interacts with multiple proteins, of which mGluR1 and mGluR5 have been most extensively studied [53] and may function as an adaptor or scaffold protein linking membrane receptors and lipids, thus facilitating receptor trafficking and/or recycling. Our study highlights the possibility that neurochondrin depalmitoylation is crucial for supporting neuronal plasticity via yet an unknown mechanism. In this regard, LTP induction is associated with increased neurochondrin expression while neurochondrin deficiency results in increased CaMKII activity in hippocampal lysates and impaired spatial learning [55]. Considering results shown in this study, we may speculate that depalmitoylation of neurochondrin at cysteines C3 and C4 prevents negative regulation of CaMKII phosphorylation and promotes CaMKII activity [8, 55, 56]. Alternatively, palmitoylation at the C-terminal region could affect an interaction of neurochondrin with proteins such as Dia1 and others (i.e. mGluR5 or CaMKII) and in this way support synaptic plasticity [57].

Neural cell adhesion molecule NCAM mediates cell-cell and cell-matrix adhesion and plays a key role in neurite outgrowth, synaptogenesis, and synaptic plasticity [58]. NCAM contains 3 cysteines that undergo palmitoylation. This modification helps NCAM target lipid rafts, facilitates NCAM-mediated signaling and neurite outgrowth [59]. It was shown previously that NCAM140 and NCAM180 are covalently modified by thioester-linked palmitate following fibroblast growth factor 2 (FGF2) - stimulated neurite outgrowth [60] and NCAM was identified as substrate for ZDHHC3 and ZDHHC7 [8]. Palmitoylation of NCAM may also help in its endocytosis and recycling to the plasma membrane [61]. A presence of NCAM blocking antibody or NCAM knockout both have negative effects on the early phase of synaptic potentiation lasting minutes [62]. In this study NCAM palmitoylation was decreased 20 min after induction of synaptic plasticity with cLTP in neuronal cultures or stimulation of synaptoneurosomes. The role of rapid NCAM depalmitoylation in supporting synaptic plasticity remains unclear. We propose that NCAM depalmitoylation could increase its mobility between compartments and reduce the proportion of NCAM anchored in the membrane toward the extracellular space. This reduction in NCAM-mediated synaptic adhesion and interactions with extracellular matrix components, including heparan proteoglycans, is thought to enhance the structural flexibility required for synaptic plasticity.

### Synaptoneurosomes and protein depalmitoylation upon LTP

One of the most surprising findings of this study is observation that exposing live, extracted excitatory synapses to a temperature change or NMDAR-dependent LTP (STIM) resulted in protein-specific dynamic palmitoylation occurring within minutes. This can’t be explained by change in protein abundance, because STIM affected only 2% of proteins, mainly ribosomal proteins and only 0.44% were non- ribosomal UniProt assigned proteins (as expected for local translation of proteins occurring in the synapse [37]). At the same time, 3.9–10.6% of all proteins exhibited altered palmitoylation levels (Data S1). Previous studies implied that in neurons several enzymes including ZDHHC1, ZDHHC2, ZDHHC5 and ZDHHC8 may be present in dendrites or synapses [8], while ZDHHC3, ZDHHC7 and ZDHHC12 were found in somatic Golgi. ZDHHC17 was additionally associated with several vesicular structures, including the Golgi apparatus as well as sorting/recycling and late endosomal structures and was found in the presynaptic terminal in drosophila [63]. In this study using mass spectrometry of synaptoneurosomes we found the presence of only two ZDHHCs (ZDHHC5 and ZDHHC17) and all known members of depalmitoylating machinery including APT1/2, PPT1, and ABHDs (see also [28]). We did not detect Golgi marker proteins in synaptoneurosomal fractions (i.e. GM130, SialT2, TGN38 (Data S1) [64]). The use of live synaptoneurosomes excludes the possibility of extensive ultracentrifugation to obtain ultrapure synaptic fraction. However, our protocol for generating live synaptoneurosomes results in a significant enrichment of proteins of excitatory synapses over inhibitory ones (Fig. S6) and a depletion of cytosolic and nuclear markers in the synaptoneurosomes fraction as compared to the homogenate [65]. Therefore palmitoylation in synaptoneurosomes could be mediated primarily by non-Golgi located ZDHHCs like ZDHHC5 while depalmitoylation enzymes seem to be overrepresented. It was previously shown that PPT1 is present in the synaptic compartment of the rat neurons and high levels of PPT1 enzyme activity was found in the synaptic cytosol [66]. In addition, approximately 10% of palmitoylated synaptic proteins were reported as a substrate of PPT1. In this study, among palmitoylated proteins we found that neural cell adhesion molecule (Nrcam), neurotrimin and sodium/potassium-transporting ATPase subunit beta-2 were significantly depalmitoylated 20 min post glycine + KCl treatment. These proteins were reported substrates to PPT1 [66]. Thus, one explanation of our results is that PPT1 activity in excitatory synapses may be triggered by external stimulation.

As an enzymatic process, palmitoylation is expected to be highly temperature-dependent, while the resulting thioester bond is covalent and relatively stable. Indeed, in our experiments, palmitoylomes of samples maintained at 4 °C versus 37 °C for 30 min displayed distinct patterns across hundreds of proteins (Fig. 6). Temperature may affect membrane fluidity, protein’s lateral mobility, palmitate accessibility and thus availability of both the substrate and palmitoylation enzymes, especially membrane-bound, to interact. In this mechanism, temperature could have an impact on membrane bound components of palmitoylation-machinery and affect palmitoylation kinetics. But how exactly temperature regulates palmitoylation remains elusive. Thus, one explanation for these results is the difference in enzymatic activity in the preparations further supporting a view that palmitoylation machinery is present in the excitatory synapses and responsive to external stimuli.

While ABE-WB in synaptoneurosomes showed massive depalmitoylation of proteins such as synaptophysin, NCAM, and PSD95 (Fig. 5), quantification by mass spectrometry did not show significant changes in abundance of palmitoylated forms of these proteins (Fig. 6, Fig. S7, Data S1). This discrepancy may arise due to significant differences between the techniques used. Western blot sensitivity depends on antibody specificity, while MS-based quantification depends on peptide coverage. MS data undergo multiple normalization steps to correct for background and sample variation, which can attenuate apparent effect sizes. MS data processing inherently includes some degree of uncertainty: for example, an FDR of 0.01 is used for protein identification, and FDR ≤ 0.05 is applied to correct for multiple hypothesis testing when assessing differences in palmitoylation levels. This approach ensures the robustness of the dataset as a whole, although it may reduce sensitivity for detecting changes in individual proteins, particularly those represented by a limited number of peptides. Additional post-translational modifications (PTMs) can affect detectability and quantification in both techniques, further complicating direct comparisons. While both ABE-WB and ABE-MS are valuable, they provide complementary perspectives. ABE-WB allows sensitive, targeted detection of specific proteins, whereas ABE-MS offers a broad, untargeted, system-level view.

### Calcium signaling and the dynamic regulation of protein palmitoylation in neuronal plasticity

It is well established that in neurons, protein palmitoylation heavily depends on neuronal activity, and intracellular cascades mediating the transduction of extracellular stimuli to palmitoylation machinery activity are beginning to be unraveled [4]. However, a key question arises: how do external stimuli translate into the control of protein palmitoylation on demand in neurons? In this study, we induced NMDAR-dependent forms of synaptic plasticity to mimic those occurring in vivo [16, 18]. NMDARs regulate Ca²⁺ entry into the postsynaptic compartment. Therefore, in a simplified view, palmitoylation may be regulated by pathways that are heavily dependent on Ca²⁺. Indeed, palmitate cycling on PSD95 was shown to be regulated by Ca²⁺ influx through NMDARs and to be absent in Ca²⁺-free solutions [67]. Additionally, NCAM was shown to interact with calmodulin, a Ca²⁺-binding protein. Translocation of NCAM to the ER and from the ER membrane to the cytoplasm, and its import from the cytoplasm to the nucleus are calmodulin- and calcium-dependent processes [68]. Moreover, external stimuli such as fibroblast growth factor 2 (FGF-2), which is known to promote LTP in the hippocampus, have been shown to induce NCAM palmitoylation [60]. Interestingly, FGF-2 regulates NMDAR-mediated Ca²⁺ entry [69]. Thus, dynamic changes in intracellular Ca²⁺ could directly translate to protein palmitoylation. However, it remains unknown how cells manage to regulate each protein individually, resulting in the complex, protein-specific patterns of palmitoylation observed in this study (e.g., neurochondrin vs. PSD95). It is crucial to further explore the molecular signals regulating protein palmitoylation in neuronal plasticity in greater detail.

Interestingly, the direction of palmitoylation for individual proteins varied depending on the experimental model, cellular fraction, and timing after LTP induction. Results were consistent across slices and synaptoneurosomes but showed mixed outcomes in neuronal cultures (Fig. 5G). A possible explanation is that the chemical LTP protocol used in the latter model is less specific as it activates multiple plasticity-related pathways such as upgrade in neuronal firing, adenylyl cyclase activity, and cAMP and cGMP levels. In contrast, glycine-induced LTP is NMDAR-dependent and more localized to synaptic sites. This suggests that the recruitment of palmitoyltransferases and thioesterases may vary depending on the molecular pathways engaged during plasticity induction. Additionally, the striking contrast between the depalmitoylation of synaptic proteins observed in synaptoneurosomes and the enhanced palmitoylation seen in slice homogenates may be attributed to differences in tissue fractionation. This, in turn, could imply that in isolated synaptic compartments, depalmitoylating activity is the predominant response to synaptic stimulation, whereas in whole-cell contexts, palmitoyltransferase activity may dominate. The next follow up study could involve the analysis of synaptoneurosomes derived from primary neuronal cultures and stimulated acute brain slices. As we show in this study, these models permit precise manipulation of the extracellular environment and could be used to investigate how various neuronal signals (neurotransmitters and neuromodulators) are translated into specific palmitoylation patterns. This approach may also help clarify how protein subcellular localization influences its susceptibility to lipidation. Furthermore, high-purity synaptoneurosomes could be obtained via ultracentrifugation, followed by further fractionation to isolate specific sub compartments, such as presynaptic boutons.

### Palmitoylation supports short-and long-term synaptic plasticity in a synapse-specific manner

In this study, we report that the deacylation agent NtBuHA disrupted long-term synaptic plasticity in excitatory synapses of apical dendrites of CA1 pyramidal neurons (Fig. 4). This observation aligns with a recent report showing that inhibition of hippocampal palmitoyl acyltransferase activity with 2-BP impairs in vivo hfsLTP in the CA1 region of the hippocampus [14]. Additionally, transgenic animals lacking ZDHHC2, ZDHHC17, or PPT1 were reported to have impaired LTP [11, 70, 71]. Thus, palmitoylation appears to be indispensable for supporting synaptic plasticity. However, we also show that in the SO, NtBuHA did not affect the time course of hfsLTP nor the short*-*term synaptic plasticity (Fig. 4). The underlying mechanisms remain elusive. Nevertheless, we previously demonstrated that while excitatory synapses in the SO and SR require NMDAR activity as a critical component for LTP induction [35], they differ significantly in the downstream molecular machinery supporting synaptic potentiation. Thus, palmitoylation may regulate only certain pathways required for NMDAR-dependent plasticity. Pyramidal neurons, with their basal and apical dendrites, play a crucial role in information processing. Future studies could benefit from investigating the differences between these two types of neighboring excitatory synapses to further elucidate this issue. For example, it would be valuable to explore why SR and SO synapses have distinct palmitoylation dependencies, using molecular inhibitors that target specific pathways or by silencing genes involved in palmitoylation machinery (i.e. ZDHHCs). Additionally, investigating palmitoylation’s role in other hippocampal connections, such as mossy fiber to CA3 pyramidal neurons, known for expressing NMDAR-independent forms of LTP, could be illuminating [20].

### Palmitoylation in network plasticity and memory formation

This study provides evidence that protein palmitoylation is crucial for the temporal reorganization of neuronal spiking in neural networks following episodes of enhanced neuronal activity (Fig. 2). Thus, protein palmitoylation not only supports local synaptic plasticity but also plays a critical role in the temporal organization of neuronal spiking in networks, ultimately contributing to engram formation. Supporting the role of palmitoylation in learning and memory, a recent study showed that infusion of 2-BP into the hippocampus disrupted the acquisition and maintenance of spatial memories, but not the recall of these memories [14]. Similarly, transgenic animals lacking ZDHHC2, ZDHHC9, or PPT1 exhibited impaired memory formation [11, 70, 71]. Conversely, learning-induced changes in the brain’s palmitoylome further support its involvement in memory processes [15, 51]. Since the depalmitoylating agent NtBuHA did not affect the basal temporal organization of spikes or spiking frequency in vitro before electrical stimulation, we did not further study depalmitoylation’s impact on neural intrinsic excitability. However, we cannot exclude the possibility that palmitoylation also affects other nonsynaptic forms of neural plasticity (reviewed in [72]). For instance, enhanced neuronal activity could lead to permanent changes in neuronal excitability, associated with altered passive membrane properties and ion channel expression, in the absence of synaptic gain [73]. Some ion channels like hyperpolarization-activated cyclic nucleotide-gated channels determine neuronal excitability and undergo palmitoylation [74].

## Materials and methods

### Primary neuronal cultures

All procedures on animals were carried out following the guidelines established and approved by the Polish Ethical Committee on Animal Research. Dissociated hippocampal cultures were prepared from cells mixed from hippocampi of 12 postnatal day 0 (P0) Wistar rats as described previously [75]. For click chemistry experiments, cells were plated on 13-mm-diameter coverslip (Karl Hecht glassware factory, Germany) coated with 50 µg/ml poly-D-lysine (Merck, Poland) and 2.5 µg/ml laminin (Roche, Poland) at a density of 80,000 cells per coverslip. For the ABE method, neurons were cultured in 6-well plates coated with 50 µg/ml poly-D-lysine at a density of 450,000 cells/well. The cultures were kept at 37 °C in 5% CO2 in a humidified incubator. The experiments were performed at 14–15 day in vitro (DIV).

### Cytotoxicity test

For verification of cytotoxicity of drugs used in this study, we used the CytoTox 96^®^ Non-Radioactive Cytotoxicity Assay (cat nr G1780, Promega Corporation, USA) and followed manufacturer's guidelines. The test relied on quantitative measurement of lactate dehydrogenase (LDH) released from cells with enzymatic assay, which results in the conversion of a tetrazolium salt (iodonitro-tetrazolium violet; INT) into a red formazan product. The intensity of color formed was proportional to the number of lysed cells. Visible wavelength absorbance data were collected at 490 nm using a standard 96-well plate reader. 50µL of hippocampal cultures media (3 independent cultures, experimental LDH release) were compared with medium obtained from wells where cells were subjected to complete lysis (maximum LDH release) or fresh medium (negative control).

$$\:\text{P}\text{e}\text{r}\text{c}\text{e}\text{n}\text{t}\:\text{c}\text{y}\text{t}\text{o}\text{t}\text{o}\text{x}\text{i}\text{c}\text{i}\text{t}\text{y}=\frac{Experimental\:LDH\:release\:\left({OD}_{490}\right)}{Maximum\:LDH\:release\:\left({OD}_{490}\right)}$$

### Synaptoneurosomes

Synaptoneurosomes were prepared as described previously [76] with modifications. Briefly, artificial cerebrospinal fluid (aCSF) (containing in mM: 87 NaCl, 2.5 KCl, 1.25 NaH_2_PO_4_, 25 NaHCO_3_, 0.5 CaCl_2_, 10 MgSO_4_, 20 glucose, 75 sucrose) was aerated with an aquarium pump for 30 min at 4 °C. Next, the pH was adjusted to 7.4 with dry ice. The buffer was supplemented with 1 × protease inhibitor cocktail cOmplete EDTA-free (Roche; 05056489001). Next, one month old Wistar rats were euthanized with cervical dislocation and hippocampi were dissected. The hippocampi were cut in halves along the transverse section then each part was homogenized in 1.5 ml of aCSF using Dounce homogenizer with 10–12 strokes. All steps were kept ice-cold to prevent stimulation of synaptoneurosomes. For each sample, homogenates from hippocampi of 2 rats were diluted in an ice-cold aCSF buffer to the final volume of 18 ml. The samples were loaded into 20 ml syringe and passed through a series of aCSF-pre-soaked nylon mesh filters with a pore-size of consecutively 100, 60, 30 and 10 μm (Nylon Net Filters; Merck Millipore) to 50 ml polypropylene tube in a cold room. The filtrate was centrifuged at 1000 × g for 15 min at 4॰C (Eppendorf S-4-72 swinging bucket rotor). The pellet was resuspended in 3 ml of ice-cold aCSF buffer with protease inhibitors and the sample was divided into 3 parts. Synaptoneurosomes were stimulated as described previously [38] with modifications resulting in three sample types: unstimulated 4॰C and 37॰C control samples and stimulated 37॰C. Briefly, the suspensions of synaptoneurosomes were centrifuged at 1000 × g for 15 min at 4॰C. The 4॰C and 37॰C control samples were resuspended in the aCSF solution (containing in mM: 125 NaCl, 2.5 KCl, 1.25 NaH_2_PO_4_, 25 NaHCO_3_, 2.5 CaCl_2_, 1.5 MgSO_4_, 20 glucose). The 4॰C control sample was left on ice for for 30 min and then frozen. The 37॰C control sample was supplemented with strychnine (25 µM) and incubated for 30 min in 37॰C with shaking and then frozen. Simultaneously, the stimulated sample was resuspended in magnesium-free aCSF solution (containing in mM: 125 NaCl, 26 NaHCO_3_, 1.6 NaH_2_PO_4_, 2.5 CaCl_2_, 5 KCl and 10 glucose), supplemented with strychnine (25 µM) and glycine (100 µM) and incubated for 10 min in 37॰C with shaking followed by application of high KCl (50 mM final concentration) for another 20 min and then frozen.

### Multi electrode arrays

Multielectrode arrays containing 60 micro-electrodes of 30 μm diameter and 200 μm spacing (60PedotMEA200/30iR-Au, Multichannel Systems, Germany) were autoclaved, covered with fetal bovine serum and left for 30 min to become hydrophilic. Following wash with sterile water, plates were coated with PDL (0.05 mg/ml) overnight at 37 °C and then coated again with laminin (20 µg/ml) and left overnight at 37 °C. Each culture was prepared from mixed hippocampal neurons obtained from 12 rat pups at postnatal day 0 plated at density 1 million cells/plate in Minimal Essential Medium. (MEM, Gibco, Thermofisher, Poland) for 2 h and subsequently cultured in Neurobasal Medium (Gibco, Thermofisher, Poland) with fluorinated ethylene-propylene membrane covers (ALA MEA sheets, ALA Scientific Instruments Inc., USA) for up to 21 days. All recordings of spontaneous neuronal network activity were made inside the incubator at 37 °C with MEA2100-Mini System (Multichannel Systems, Germany). Recording electrodes were considered active if spiking frequency was > 0.1 Hz. Only plates with > 60% of active electrodes were considered for further experiments. Signals were band-pass filtered at 300–5000 Hz and digitized at 25 kHz. All recordings were made in 5 min-long epochs every 15–30 min. Stimulation of the electrodes on the MEA was performed with AT-IN protocol as described previously [77]. Briefly, following basal activity recordings, stimulation was applied on a pair of two active electrodes recording neuronal spiking in two distant areas of the plate. A low-frequency stimulation consisting of 120 stimuli at 1 Hz was applied to one single electrode and theta burst stimulation (5 stimuli applied at 100 Hz, every 50 ms) to another electrode. Single pulse was applied in phase with the burst and such associative stimulation was applied for 2 min. Each pulse was bi-phasic, ± 800 mV (positive phase first) and lasted for 500 µs. Spontaneous neuronal activity was recorded for 60 min post stimulation. Spike detection was set at 6 times standard deviation of noise. Data analysis was performed in software provided by the manufacturer and custom program written in Python.

### Acute brain slices and electrophysiology

Acute hippocampal brain slices from 1.5 to 2-month-old rats were prepared according to the protocol described previously. Briefly, hippocampi from one hemisphere were dissected and cut into 350 μm thick slices using a vibratome (5100mz, Campden Instruments, USA) in oxygenated (95% O_2_, 5% CO_2_) ice-cold buffer with pH = 7.4 containing in mM: 92 NMDG, 2.5 KCl, 1.2 NaH_2_PO_4_, 30 NaHCO_3_, 0.5 CaCl_2_, 10 MgSO_4_ · 7 H_2_O, 20 HEPES, 25 glucose, 2 thiourea, 5 sodium ascorbate, 3 sodium pyruvate. Slices were recovered in solution containing in mM: 92 NaCl, 2.5 KCl, 1.2 NaH_2_PO_4_, 30 NaHCO_3_, 2 CaCl_2_, 2 MgSO_4_ · 7 H_2_O, 20 HEPES, 25 glucose, 2 thiourea, 5 sodium ascorbate, 3 sodium pyruvate for 15 min (32 °C). Finally, slices were stored in oxygenated (95% O_2_, 5% CO_2_) artificial cerebrospinal fluid (aCSF) that contained in mM: 124 NaCl, 2.5 KCl, 1.2 NaH_2_PO_4_, 24 NaHCO_3_, 2 CaCl_2_, 2 MgSO_4_ · 7 H_2_O, 5 HEPES, 12.5 glucose, pH 7.4. Recordings were made in aCSF after 2 h of slice recovery. Test stimuli (0.3 ms, 0.1 Hz) were delivered using a stimulator (A385, World Precision Instruments, USA) and a concentric bipolar electrode (FHC, Bowdoin, ME USA) placed either in SR or SO. fEPSPs were recorded with glass micropipettes that were filled with aCS*F (*1–3 MΩ resistance). Input–output (I–O) relationships were built for fEPSPs amplitudes upon monotonically increasing the stimuli in the range of 0–300 µA (13 points, applied once at 0.1 Hz). Baseline stimulation was set at 0.1 Hz, and for baseline and paired-pulse stimulation protocols (interstimulus interval 50 ms), the stimulation strength was set to 40% of the maximum fEPSP amplitude. Signals were acquired with Sutter Double IPA amplifier (Sutter Instruments, USA).

### Acyl biotin exchange method

To analyze changes in protein palmitoylation levels acyl-biotin exchange assay (ABE) was used. To extract protein from rat hippocampal tissue slices, synaptoneurosomes or primary hippocampal neurons at 14 DIV the material was lysed and homogenized using Dounce homogenizer in a buffer containing in mM: 50 Tris HCl (pH 7.5), 150 NaCl, 1 EDTA and 4% SDS, 1% Triton X-100. Proteins were reduced with 10 mM TCEP (tris(2-carboxyethyl)phosphine) for 30 min at room temperature (RT) and subsequently, the samples were incubated for 16 h at 4 °C with 50mM N-ethylmaleimide (NEM) to block free thiol groups. Next, proteins were precipitated using chloroform-methanol extraction and subsequently washed three times with 96% ethanol to remove unreacted NEM. The pellets were resuspended in the same buffer as used previously. The fraction of each sample was moved to a separate tube to create pooled-within-condition negative control samples. The remaining volume underwent the acyl-biotin exchange reaction (‘positive samples’). Both positive and negative control samples were treated with 400 µM thiol-reactive biotinylation reagent HPDP-biotin (N-[6-(biotinamido)hexyl]−30-(20-pyridyldithio)propionamide) for 1.5 h at RT. Simultaneously, only the positive samples were treated with 1 M hydroxylamine to cleave thioester-linked palmitoyl moieties and expose newly formed-thiols to HPDP-biotin. Subsequently, proteins were precipitated with chloroform-methanol method, washed three times with ethanol, and re-dissolved in a buffer with a lower detergent content (containing in mM: 50 Tris HCl (pH 7.5), 150 NaCl, 1 EDTA and 0.5% SDS, 0.2% Triton X-100). At this stage the samples were subjected to SDS-PAGE and Western blotting to analyze the global state of palmitoylation (Stain-free total protein visualization was used as a loading control) or processed further in order to isolate enriched palmitoylated protein fraction. To this end, equal amounts of protein were taken from each sample and incubated with Pierce™ High Capacity NeutrAvidin™ Agarose beads for 2 h at RT. Subsequently, the beads were washed 6 times with a buffer containing in mM: 50mM Tris (pH7.7), 600 NaCl, 1 EDTA). To elute the enriched palmitoylated proteins fraction the beads were incubated with 50 mM Tris buffer (pH 7.7) with 1% β-mercaptoethanol for 1.5 h at 37 °C. Finally, aliquots of the samples were subjected to SDS-PAGE and Western blotting to visualize and analyze palmitoylation level changes to individual proteins.

### Western blot analysis

Protein concentration in the samples was determined using a Pierce™ BCA protein Assay kit (Thermo Scientific). The samples of equal protein content or equal volume (in case of isolated enriched palmitoylated protein fraction) were supplemented with a 6x Laemmli sample buffer and heated at 95 °C for 5 min. Subsequently, the samples were subjected to a standard SDS-PAGE in TGX Stain-Free FastCast Acrylamide 10% gels (Bio-Rad) and then transferred to PVDF membranes (Immobilon-FL, Millipore) using an electrophoretic transfer system (Trans-Blot Turbo, Bio-Rad). The non-specific binding sites were blocked with either 10% skimmed milk or EveryBlot Blocking Buffer (Bio-Rad) for 1 h at RT. Then, the blots were incubated overnight at 4 °C with the primary antibodies (Table below). After incubation, the blots were washed 5x with TBS-T and subsequently incubated with secondary antibodies for 1.5 h at RT. Blots were visualized using ECL substrate (Clarity and Clarity Max, Bio-rad) and scanned using a Chemidoc image analyzer (Bio-Rad). The molecular weights of immunoreactive bands were estimated on the basis of the migration of molecular weight markers (Thermo Fisher Scientific). Signal intensity analysis was conducted using ImageLab software (Bio-Rad).

**Table:** 

Antibodies				
Target protein	Catalogue number	Blocking agent	Dilution	Manufacturer
Synaptophysin	MAB329	EveryBlot™ blocking buffer	1:2500	Merck
SNAP-25	111 004	EveryBlot™ blocking buffer	1:2500	SYSY antibodies
Phospho-p44/42 MAPK (Erk1/2)	4377 S	Milk	1:1000	Cell Signalling
PSD95	ab18258	Milk	1:2500	Abcam
Arc (Arc/Arg3.1)	156 003	Milk	1:1000	SYSY antibodies
NCAM	A309700	Milk	1:1000	Antibodies.com
Neurochondrin	A53729	Milk	1:500	Antibodies.com
VAMP2	ab3347	EveryBlot™ blocking buffer	1:1000	Abcam
GluR1	ABN241	EveryBlot™ blocking buffer	1:1000	Merck
Gephyrin	ab32206	Milk	1:1000	Abcam

### Click chemistry

Neuronal cell cultures were grown on coverslips and on 13 DIV received alkyne-palmitic acid (50 µM) dissolved in dimethyl sulfoxide (Sigma Aldrich). Additionally, some cells received NtBuHA dissolved in water (1 mM) or solvent and were incubated overnight. On 14 DIV cells were washed three times with phosphate-buffered saline (PBS), fixed in cold methanol for 10 min at −20 °C, and permeabilized in 0.1% Triton X-100 in PBS for 5 min. The click-chemistry reaction involved 1 h incubation at room temperature with a mixture of Oregon Green 488 azide (0.1 mM) (Thermo Fisher Scientific), TCEP (1 mM), and CuSO4 (0.1 mM). Next, the cells were washed five times with PBS, and the coverslips were mounted in Fluoromount G anti-quenching medium. Images of neurons were acquired using a Zeiss LSM780 laser scanning confocal microscope with a Plan Apochromat 63×/1.4 oil immersion objective using a 488 nm diode-pumped solid-state laser at a pixel count 1024 × 1024 at 16 bit depth. A series of z-stacks were collected for each stimulation with a 1 μm step size. The fluorescence intensity was determined using Fiji open source image processing package [78].

### Proteomic sample preparation, LC-MS/MS measurements, and data analysis

INPUT SAMPLES were subjected to chloroform/methanol precipitation, and the resulting protein pellets were washed twice with methanol. The protein pellets were air-dried for ~ 10 min and resuspended in 100 mM HEPES (pH 8) by vigorous vortexing and sonication. Proteins were then reduced and alkylated with 10 mM tris(2-carboxyethyl)phosphine hydrochloride (TCEP)/40 mM chloroacetamide (CAA) and digested with trypsin in a 1:30 (w/w) enzyme-to-protein ratio at 37 °C overnight. Digestion was terminated by the addition of trifluoroacetic acid (TFA) to a 1% final concentration. The resulting peptides were labelled using an on-column TMT labelling protocol [79]. TMT-labelled samples were compiled into a single TMT sample and concentrated using a SpeedVac concentrator. Peptides in the compiled sample were separated into fractions with basic reversed-phase using the Pierce High pH Reversed-Phase Peptide Fractionation Kit (Thermo Fisher Scientific). PALM SAMPLES were dried using a SpeedVac concentrator, then resuspended in 100 mM HEPES (pH 8) by vigorous vortexing and sonication. Proteins were reduced and alkylated with 10 mM TCEP/40 mM CAA and digested with trypsin in a 1:30 (w/w) enzyme-to-protein ratio at 37 °C overnight. Digestion was terminated by the addition of trifluoroacetic acid (TFA) to a 1% final concentration. The resulting peptides were labelled using an on-column TMT labeling protocol. TMT-labelled samples were compiled into a single TMT sample and concentrated using a SpeedVac concentrator. Prior to the liquid chromatography (LC)-MS measurement, the peptide fractions were resuspended in 0.1% TFA and 2% acetonitrile in water. Chromatographic separation was performed on an Easy-Spray Acclaim PepMap column (50 cm length × 75 μm inner diameter; Thermo Fisher Scientific) at 55 °C by applying 120 min acetonitrile gradients in 0.1% aqueous formic acid at a flow rate of 300 nl/min. An UltiMate 3000 nano-LC system was coupled to a Q Exactive HF-X mass spectrometer via an easy-spray source (all Thermo Fisher Scientific). The Q Exactive HF-X was operated in TMT mode with survey scans acquired at a resolution of 60,000 at m/z 200. Up to 15 of the most abundant isotope patterns with charges 2–5 from the survey scan were selected with an isolation window of 0.7 m/z and fragmented by higher-energy collision dissociation with normalized collision energies of 32, while the dynamic exclusion was set to 35 s. The maximum ion injection times for the survey scan and dual MS (MS/MS) scans (acquired with a resolution of 45,000 at m/z 200) were 50 and 120 ms, respectively. The ion target value for MS was set to 3e6 and for MS/MS was set to 1e5, and the minimum AGC target was set to 1e3. Prior to the liquid chromatography (LC)-MS measurement, the peptide fractions were resuspended in 0.1% TFA and 2% acetonitrile in water. Chromatographic separation was performed on an Easy-Spray Acclaim PepMap column (50 cm length × 75 μm inner diameter; Thermo Fisher Scientific) at 55 °C by applying 120 min acetonitrile gradients in 0.1% aqueous formic acid at a flow rate of 300 nl/min. An UltiMate 3000 nano-LC system was coupled to a Q Exactive HF-X mass spectrometer via an easy-spray source (all Thermo Fisher Scientific). The Q Exactive HF-X was operated in TMT mode with survey scans acquired at a resolution of 60,000 at m/z 200. Up to 15 of the most abundant isotope patterns with charges 2–5 from the survey scan were selected with an isolation window of 0.7 m/z and fragmented by higher-energy collision dissociation with normalized collision energies of 32, while the dynamic exclusion was set to 35 s. The maximum ion injection times for the survey scan and dual MS (MS/MS) scans (acquired with a resolution of 45,000 at m/z 200) were 50 and 120 ms, respectively. The ion target value for MS was set to 3e6 and for MS/MS was set to 1e5, and the minimum AGC target was set to 1e3. The data were processed with MaxQuant v. 1.6.17.0 and the peptides were identified from the MS/MS spectra searched against Uniprot Rat Reference Proteome (UP000002494) using the buil*t-*in Andromeda search engine. Raw data related to PALM SAMPLES and INPUT SAMPLES were processed together using MaxQuant software. Reporter ion MS2-based quantification was applied with reporter mass tolerance = 0.003 Da and min. reporter PIF = 0.75. Cysteine carbamidomethylation was set as a fixed modification and methionine oxidation, glutamine/asparagine deamidation as well as protein N-terminal acetylation were set as variable modifications. For in silico digests of the reference proteome, cleavages of arginine or lysine followed by any amino acid were allowed (trypsin/P), and up to two missed cleavages were allowed. The FDR was set to 0.01 for peptides, proteins and sites. Match between runs was enabled. Other parameters were used as pre-set in the software. Reporter intensity corrected values for protein groups were loaded into Perseus v. 1.6.10. Standard filtering steps were applied to clean up the dataset: reverse (matched to decoy database), only identified by site, and potential contaminant (from a list of commonly occurring contaminants included in MaxQuant) protein groups were removed. Reporter intensity corrected values were log2 transformed and protein groups with values for all PALM samples were kept. The values recorded for the PALM samples not treated with hydroxylamine were subtracted from the values recorded for the PALM samples subjected to hydroxylamine treatment. Values recorded for the INPUT samples were normalized by median subtraction within TMT channels. The values recorded for the INPUT samples were subtracted from the values recorded for the corresponding PALM samples. Median was then subtracted within TMT channels. To determine which proteins were differentially palmitoylated following temperature shift/stimulation. Student’s *t-*tests (2-sided, permutation-based FDR = 0.05, S0 = 0.1, *n* = 4) were performed on the groups of PALM samples. In addition, Student’s *t-*tests (2-sided, permutation-based FDR = 0.05, S0 = 0.1, *n* = 4) were performed on the groups of INPUT samples to determine possible changes in protein levels following temperature shift/stimulation. For lists of proteins identified and quantified in these experiments, see Data S1. This dataset was deposited to the ProteomeXchange Consortium via the PRIDE partner repository with the dataset identifier PXD058417. To characterize palmitoylated proteins in rat hippocampal synaptoneurosomes we used TMT-based quantitative proteomics. KEGG and GO functional protein enrichment analyses were performed using the web-based tool ShinyGO 0.81. The 116 differentially palmitoylated proteins identified in synaptoneurosomes following KCl stimulation (mapped to 101 unique rat genes, or 87%) were queried against the Rattus norvegicus hippocampal proteome database, specifically proteins identified with TMT labeling (6,359 proteins [80]). Pathway enrichment analysis (KEGG) and enrichment for GO molecular function, cellular component, and biological process terms were Limited to top 20 results. Enriched pathways were ordered by the negative logarithm of their p-value. All significant GO terms with *p <* 0.05 (corrected for multiple testing using the Benjamini-Hochberg false discovery rate method) were selected as overrepresented.

## Supplementary Information

Below is the link to the electronic supplementary material.

Supplementary Material 1 (PDF 3.45 MB)

Supplementary Material 2 (XLSX 1.78 MB)

## Data Availability

All data are available in the main text or the supplementary materials.
