# Supplementary material for: Temporal and protein-specific S-palmitoylation supports synaptic and neural network plasticity
Source: Cell Mol Life Sci. 2025 Oct 11;82(1):355. doi: 10.1007/s00018-025-05893-5 (PMC12515174; doi:10.1007/s00018-025-05893-5)
Supplement: Supplementary file 1 — (PDF 3.45 MB) [file 18_2025_5893_MOESM1_ESM.pdf]

Temporal and protein-specific S-palmitoylation supports synaptic and neural network plasticity

Cellular and Molecular Life Sciences

Agata Pytyś[1,2], Rabia Ijaz[1], Anna Buszka[1], Jacek Miłek[3], Izabela Figiel[1], Patrycja Wardaszka[3], Matylda Roszkowska[4], Natalia Mierzwa[1], Adam Wojtas[1], Eli Kerstein[5], Remigiusz Serwa[6], Katarzyna Kalita[4], Rhonda Dzakpasu[5], Magdalena Dziembowska[3], Jakub Włodarczyk[1], Tomasz Wójtowicz[1,2*]

[1] Laboratory of Cell Biophysics, Nencki Institute of Experimental Biology of the Polish Academy of Sciences; Warsaw, Poland

[2] Laboratory of Molecular Basis of Behavior, Nencki Institute of Experimental Biology of the Polish Academy of Sciences; Warsaw, Poland

[3] Department of Animal Physiology, Faculty of Biology, University of Warsaw; Warsaw, Poland

[4] Laboratory of Neurobiology, BRAINCITY, Nencki Institute of Experimental Biology of the Polish Academy of Sciences; Warsaw, Poland

[5] Department of Physics, Georgetown University; Washington, D.C., United States of America

[6] Proteomics Core Facility, The International Institute of Molecular Mechanisms and Machines of the Polish Academy of Sciences; Warsaw, Poland

*Corresponding author. Email: t.wojtowicz@nencki.edu.pl

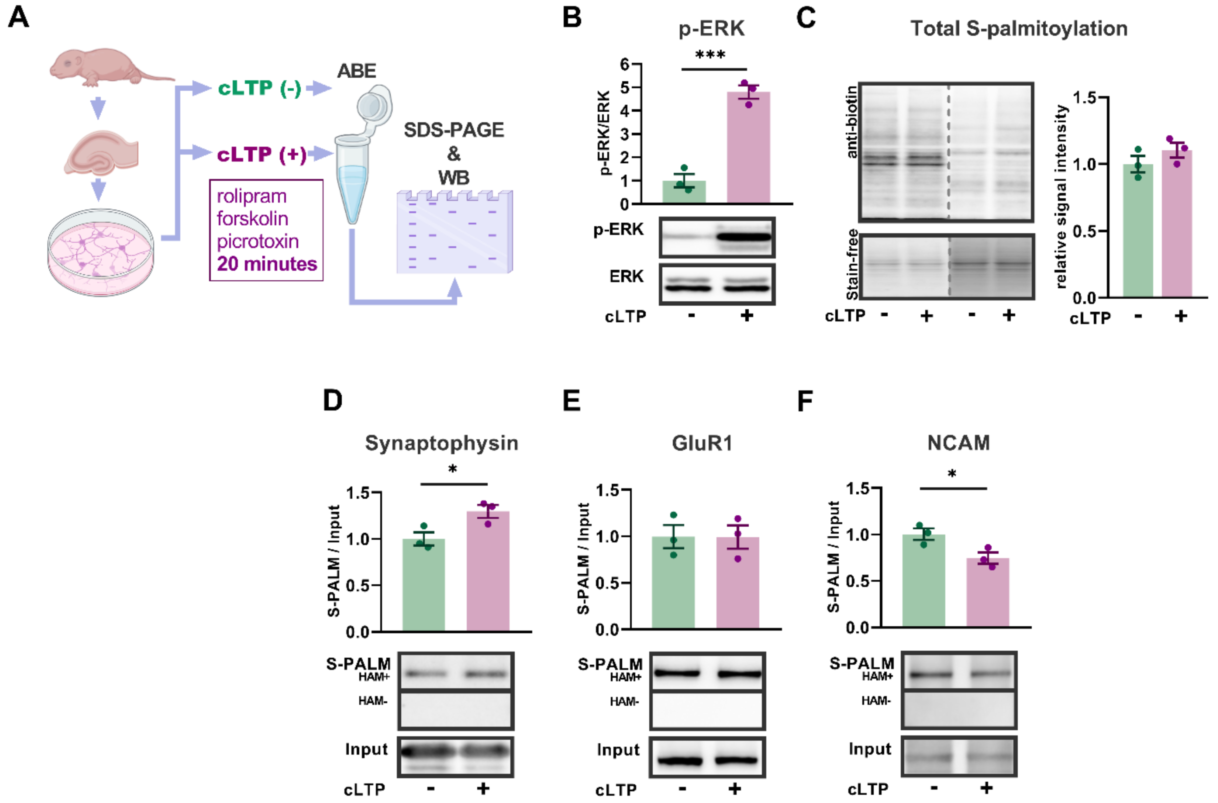

Fig S1. Induction of synaptic plasticity in neuronal cultures regulates palmitoylation of synaptic proteins. (A) Primary rat hippocampal neurons were cultured for 14 days and treated with a cLTP cocktail containing rolipram, forskolin and picrotoxin (cLTP +) or solvent (cLTP -). Culture homogenates were collected 20 min after treatment, subjected to ABE and S-PALM and input fractions were immunoblotted for target proteins, as indicated. (B) Quantification of the phosphorylated extracellular regulated kinase (pERK) protein expression in culture homogenates 20 min post cLTP (note a significant increase in the expression of pERK protein, n = 3 cultures, each culture was prepared from mixed hippocampal neurons of N = 12 animals, see Materials and Methods section, $p = 0.0007$, unpaired Student's t-test) (C) Western blot of global palmitoylation 20 min post cLTP indicated no shift in global protein palmitoylation (n = 3 cultures, each culture was prepared from mixed hippocampal neurons of N = 12 animals, see Materials and Methods section, p = 0.27, unpaired Student's t-test). (D-F) Western blot and quantification of the ABE assay described in (A) performed on exemplary presynaptic (D), postsynaptic (E) proteins and extracellular synaptic adhesion molecules. The levels of palmitoylated synaptophysin and NCAM increased significantly post cLTP ($n = 3$ cultures, $p = 0.039$ and $p = 0.041$, respectively, unpaired Student's t-test). Data are means ± SEM. *$p < 0.05$.

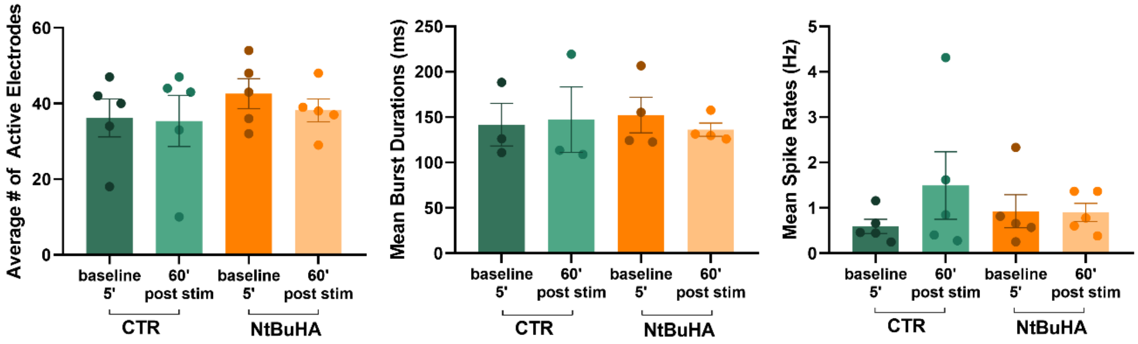

Fig S2. Protein deacylation does not affect neuronal spiking *in vitro*. Primary rat hippocampal neurons were cultured for 14 days on multielectrode arrays (MEAs), enabling recordings of action potentials from up to 60 network sites. Spikes were quantified in control cultures (green) and cultures treated overnight with NtBuHA (orange) before and after inducing network plasticity through associative activity (stim.). There were no significant main effects of the drug or stimulation on the number of active electrodes, burst duration, or mean spike firing rate ($F_{(1,8)}$ = 0.135, p = 0.71; $F_{(1,10)}$ = 0.24, p = 0.63; $F_{(1,16)}$ = 1,15, p = 0.29, two-way ANOVA, treatment x stimulation). Data are shown as mean ± SEM. n = 5 MEAs from 3 separate cultures per group.

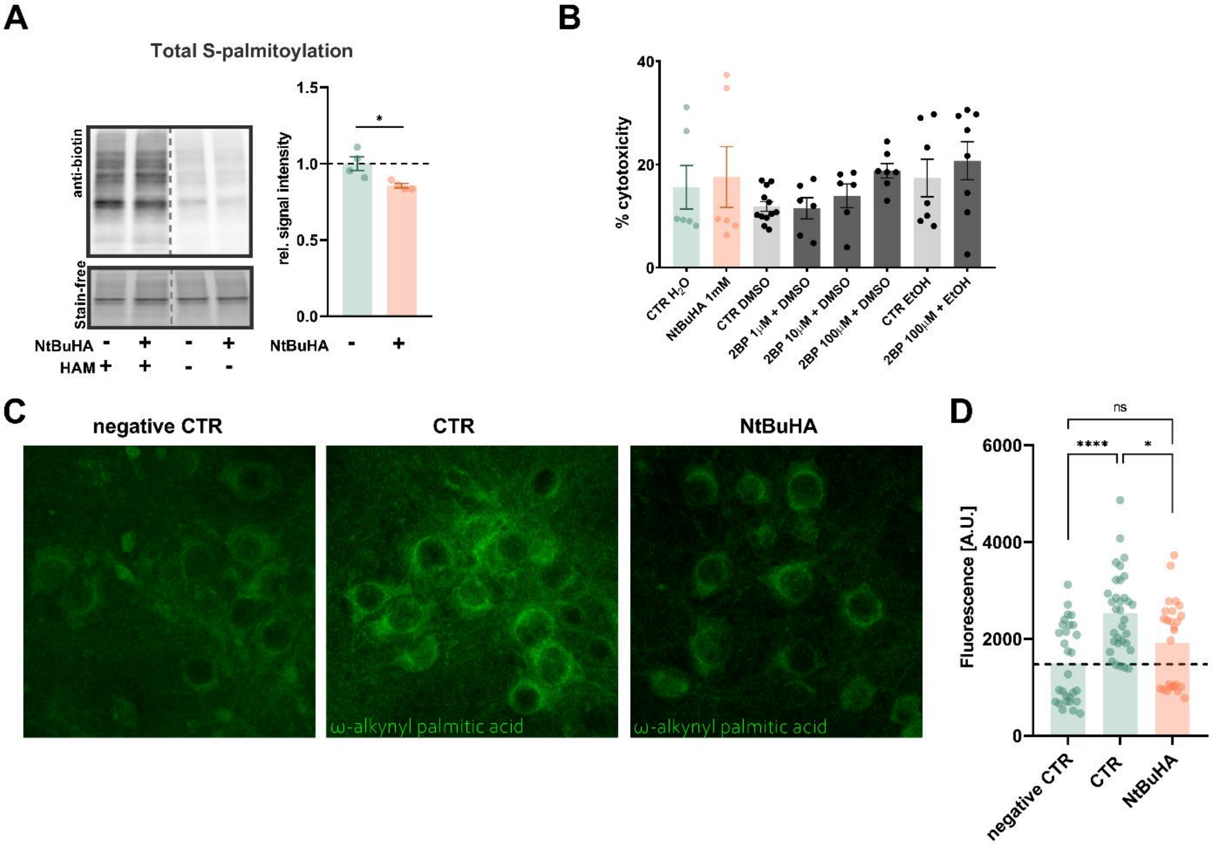

Fig S3. Deacylation of proteins in primary rat hippocampal cultures with NtBuHA. (A) Cultures at 14 DIV were treated overnight with 1 mM NtBuHA (each culture was established from mixed neurons of 12 Wistar P0 rat pups, see Methods). Global protein palmitoylation was assessed using HRP-streptavidin on immunoblotted samples of whole-culture homogenates after the acyl-biotin exchange (ABE). HAM stands for hydroxylamine. Left panel shows an exemplary Western blot and the right panel shows the quantification of all experiments. NtBuHA significantly reduced S-PALM of proteins when normalized to the loading control (stain-free total protein intensity; $n = 4$ cultures; $p = 0.021$, unpaired Student's *t*-test). (B) Primary hippocampal cultures at 14 DIV were treated overnight with 1 mM NtBuHA (dissolved in water) or with 2BP (1–100 µM) dissolved in DMSO or ethanol. Culture media were collected and analyzed for lactate dehydrogenase (LDH) release using the CytoTox 96 Cytotoxicity Assay. Treatments did not significantly affect cytotoxicity levels ($n \geq 6$ cultures; one-way ANOVA, $F_{(7, 50)} = 1.291$, $p = 0.27$). (C) Primary rat hippocampal cultures at 14 DIV were treated overnight with (CTR and NtBuHA) or without exogenous alkynyl palmitic acid (negative CTR). Exemplary images show fluorescent labeling of exogenously applied palmitate in neuronal cultures, following a click chemistry reaction with azide-tagged Oregon Green 488 dye. Additionally, 1 mM NtBuHA was added to some cultures. (D) Quantification of experiments described in (C). Control cultures had significantly larger fluorescence compared to negative CTR and NtBuHA treated cultures (n = 4-11 cover slips per culture, each photographed in 3 individual locations and averaged, N = 3 cultures, see Materials and Methods section). $F_{(2, 85)} = 11.80$, $p < 0.0001$, one-way ANOVA with Tukey post-hoc). Data are means ± SEM. Asterisks indicate statistical significance: * $p < 0.05$, **** $p < 0.0001$.

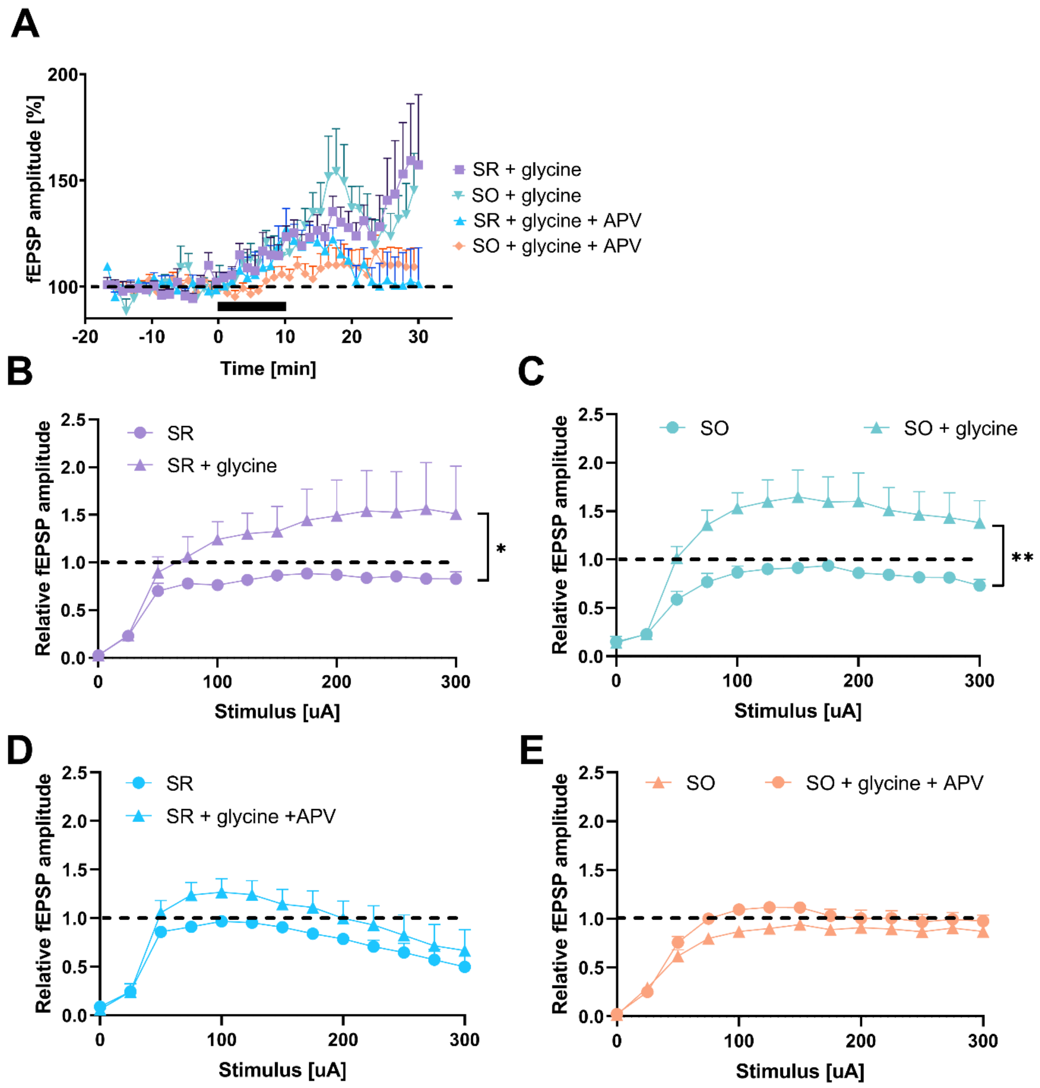

Fig S4. Glycine-induced NMDAR-dependent synaptic potentiation in hippocampal slices.

(A) Time course of fEPSPs recorded in stratum radiatum (SR) or stratum oriens (SO) of CA1 hippocampal region in response to extracellular stimulation of Schaffer collaterals. Addition of glycine (600µM, at time 0 min. for 10 minutes) in Mg^{2+}-free aCSF resulted in an enhancement of fEPSP amplitudes both in SR and SO (indicated with black rectangle). In some experiments NMDAR antagonist APV (50µM) was applied (n = 6 - 8 slices, N = 3 - 4 animals per group). (B-E) Statistical analysis of fEPSP amplitudes recorded in response to a wide range of stimuli before and 30 minutes after application of glycine in slices shown in A. (B, C) Glycine significantly enhanced fEPSP amplitudes in SR (B) (n = 8 slices, N = 4 animals, $F_{(12, 168)}$ = 1.831, p = 0.04, two-way ANOVA, treatment × stimulus intensity) and in SO (C) (n = 6 slices, N = 3 animals, $F_{(12, 120)}$ = 3.432, p = 0.0002, two-way ANOVA, treatment × stimulus intensity). The potentiating effect of glycine was not observed in the presence of APV in both SR (D) (n = 7 slices, N = 3 animals, $F_{(12, 144)}$ = 0.86, p = 0.58, two-way ANOVA, treatment × stimulus intensity) and in SO (E) (n = 8 slices, N = 3 animals, $F_{(12, 168)}$ = 1.719, p = 0.066, two-way ANOVA, treatment × stimulus intensity). Asterisks indicate statistical significance: * p < 0.05, ** p < 0.01.

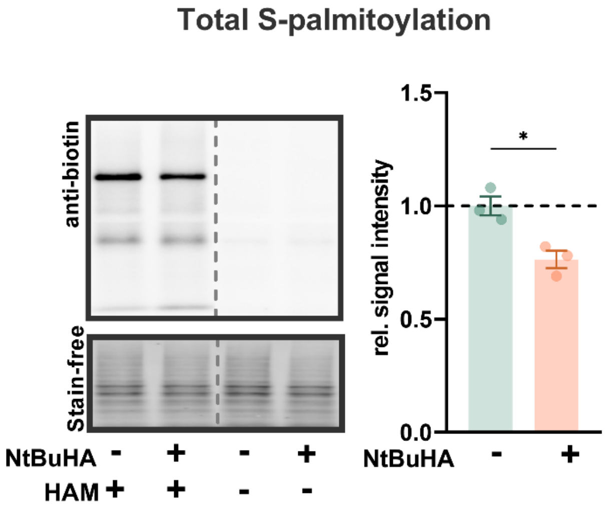

Fig S5. Deacylation of proteins in hippocampal slices with NtBuHA. Hippocampi from Wistar rats aged 45-60 days were cut in slices (350µm thick) and left in either aCSF or aCSF containing NtBuHA (1mM) for 2 hours. Global protein palmitoylation was assessed using HRP-streptavidin after the acyl-biotin exchange (ABE) protocol on tissue homogenates. HAM stands for hydroxylamine. The left panel shows an exemplary Western blot and the right panel shows the quantification of all experiments. NtBuHA significantly reduced S-palmitoylation of proteins when normalized to the loading control (stain-free total protein intensity; n = 3 animals; p = 0.014, unpaired Student's t-test). Asterisks indicate statistical significance: * $p < 0.05$.

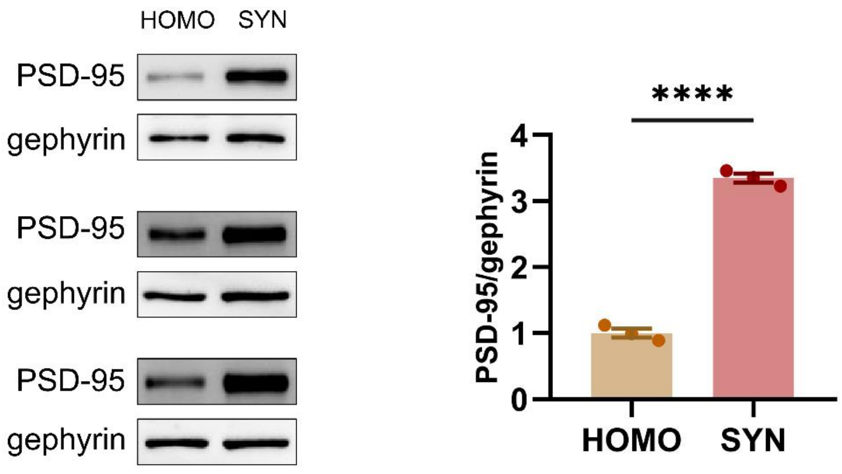

Fig S6. Live synaptoneurosomes exhibit significant enrichment in PSD95 but not gephyrin. Left panel shows Western blots (WB) for PSD-95 and gephyrin obtained in hippocampal homogenates (HOMO) and synaptoneurosomes (SYN) from young Wistar rats (for each sample, homogenates were obtained from hippocampi of 2 rats). Right panel shows the quantification of WB. Live synaptoneurosomes exhibit significant enrichment in PSD-95 (n = 3 samples, N = 6 animals; **$p < 0.0001$**, unpaired Student's t-test). Asterisks indicate statistical significance: ****** $p < 0.0001$**. **Full images of the blots, including molecular weight markers, are provided in Supplementary Figure S8.**

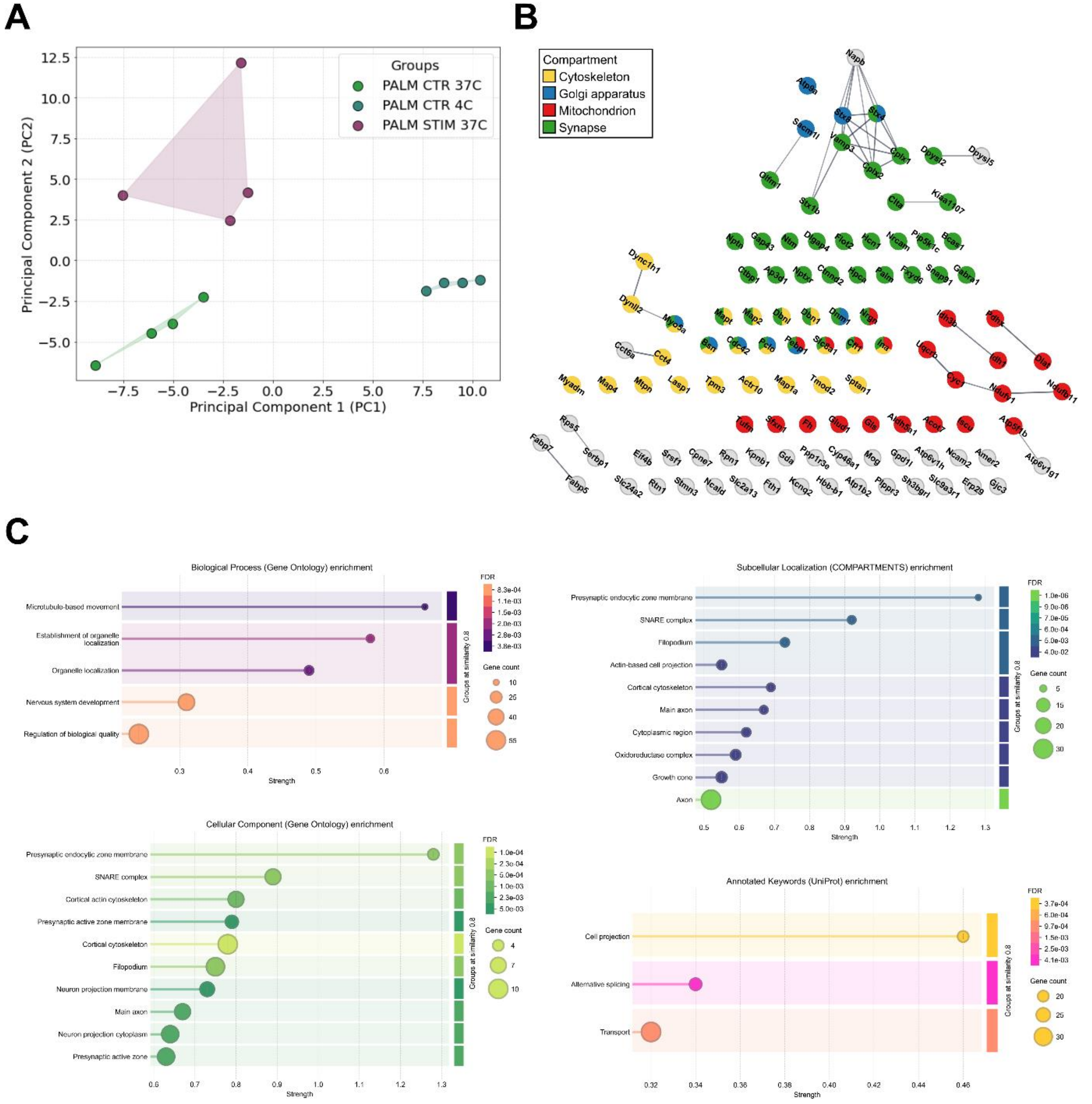

Fig S7. STRING-based protein-protein interaction (PPI) network analysis of differentially palmitoylated proteins in synaptoneurosomes following stimulation with glycine + KCl (protein names have been converted to corresponding gene names throughout the figure for clarity and ease of presentation).

(A) Principal component analysis (PCA) of palmitoylated protein profiles in hippocampal synaptoneurosomes following stimulation with KCl (50 mM) and glycine (100 µM) in Mg^{2+}-free solution (PALM STIM 37°C group). Control samples (PALM CTR 37°C and PALM CTR 4°C) were incubated without these agents but in the presence of Mg^{2+} (n = 4 samples per group; for each sample, homogenates

from hippocampi of 2 rats were used). Note that cooling or stimulation of synaptoneurosomes resulted in separation and clustering of the proteomic profiles between experimental groups. (B) STRING database (https://string-db.orsg) was used for network analysis of 117 proteins differentially palmitoylated between the PALM STIM 37°C and PALM CTR 37°C groups. Direct (physical) protein-protein interactions are shown, with a minimum required interaction score set to high confidence (≥0.7). Each node represents a protein; edges indicate known or predicted interactions based on text mining, experimental data, and curated databases. The full dataset of 4,502 proteins detected in input samples was used as background for enrichment analysis.
(C) STRING visualization of category enrichment within the dataset, highlighting biological processes and pathways significantly associated with the differentially palmitoylated proteins in the PALM STIM 37°C group.

Fig S8. Western blots for palmitoyl-proteins and neuronal plasticity markers quantified in this study. Western blots are presented as composite images, combining the chemiluminescence channel with the white-light channel (membrane image with the molecular weight marker). Some lanes were excluded from quantification because they were irrelevant to the study (e.g., samples collected at time points not represented in the figure or compromised due to weak signal, rendering them unquantifiable).

ABE RESULTS

NEURONAL CULTURES

FIG. 1B AND FIG. S1B

replicate shown in the main figure

pERK/ERK

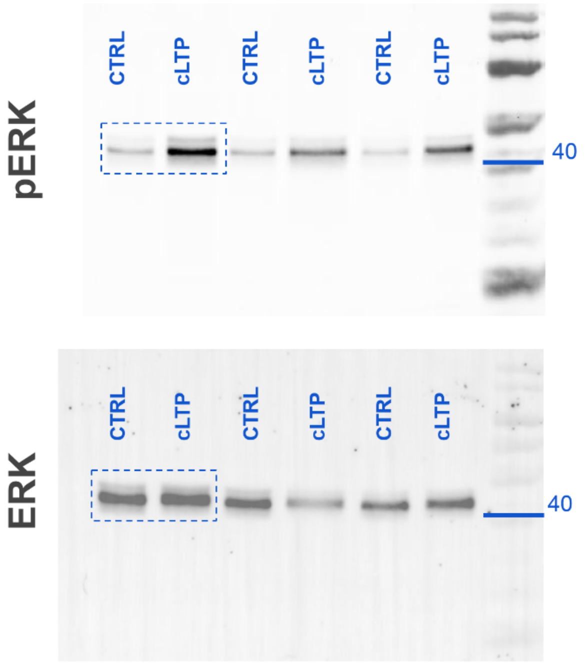

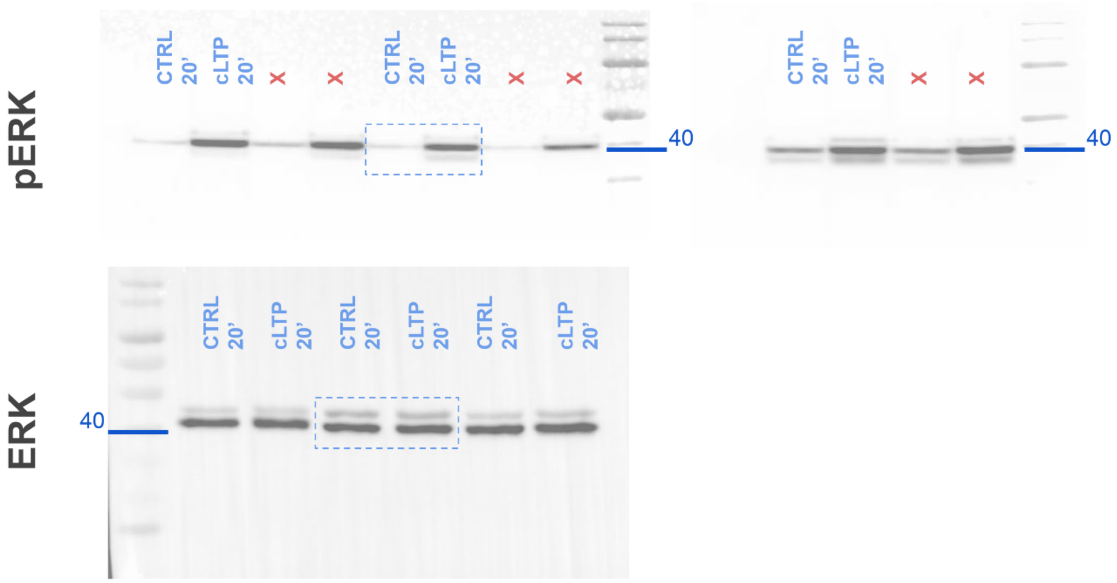

ABE RESULTS

NEURONAL CULTURES

FIG. 1B AND FIG. S1B

replicate shown in the main figure

pERK/ERK

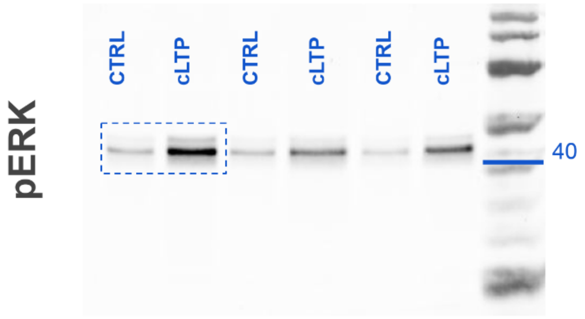

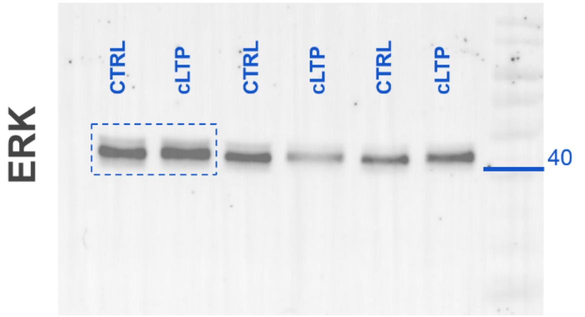

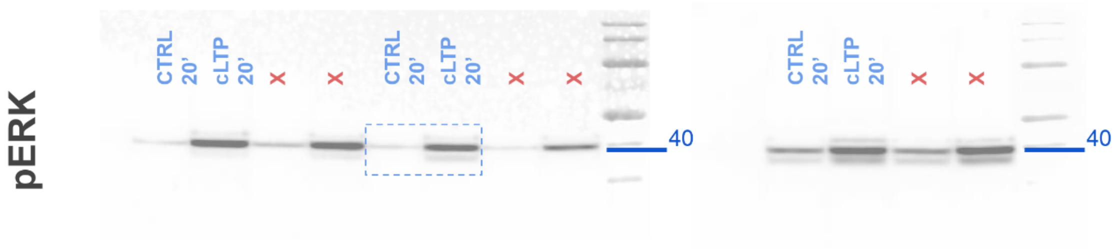

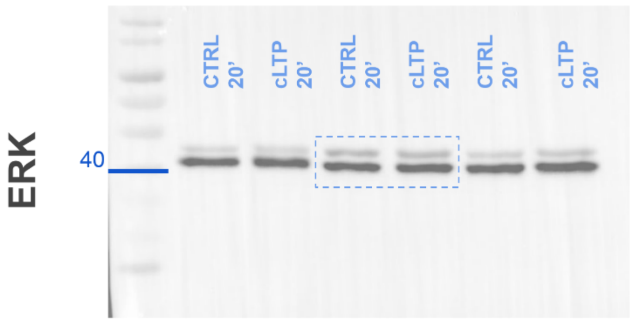

ABE RESULTS

NEURONAL CULTURES

FIG. 1B AND FIG. S1B

replicate shown in the main figure

pERK/ERK

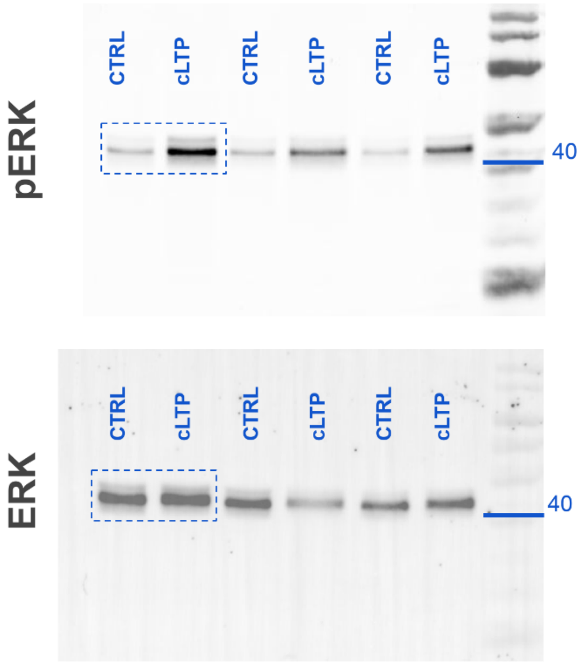

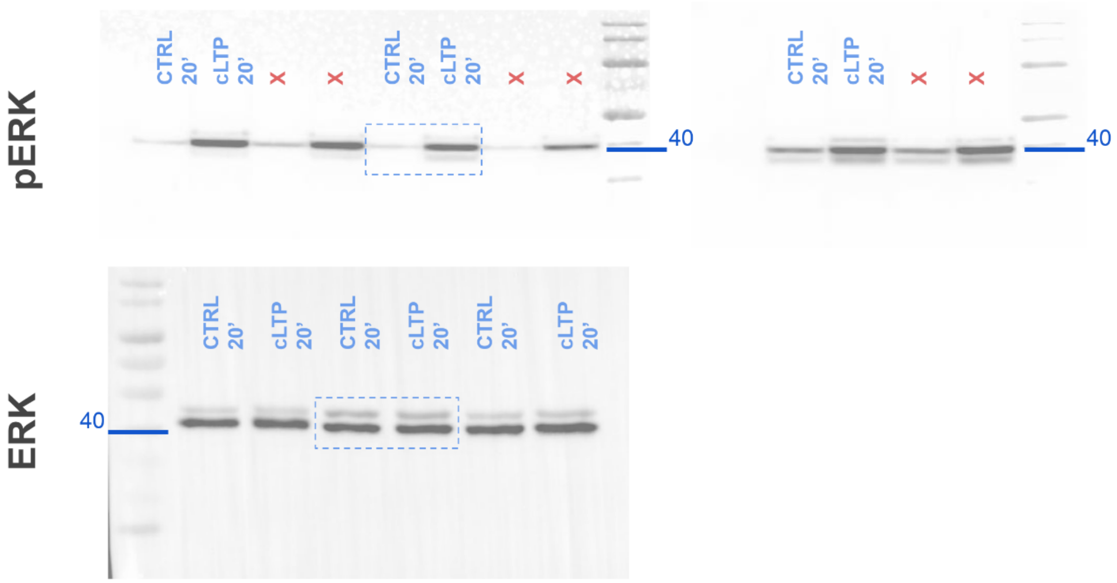

ABE RESULTS

NEURONAL CULTURES

FIG. 1D AND FIG. S1D

positive results (HAM+)
negative controls (HAM-)
× irrelevant lanes (not included in the results)
replicate shown in the main figure

SYNAPTOPHYSIN

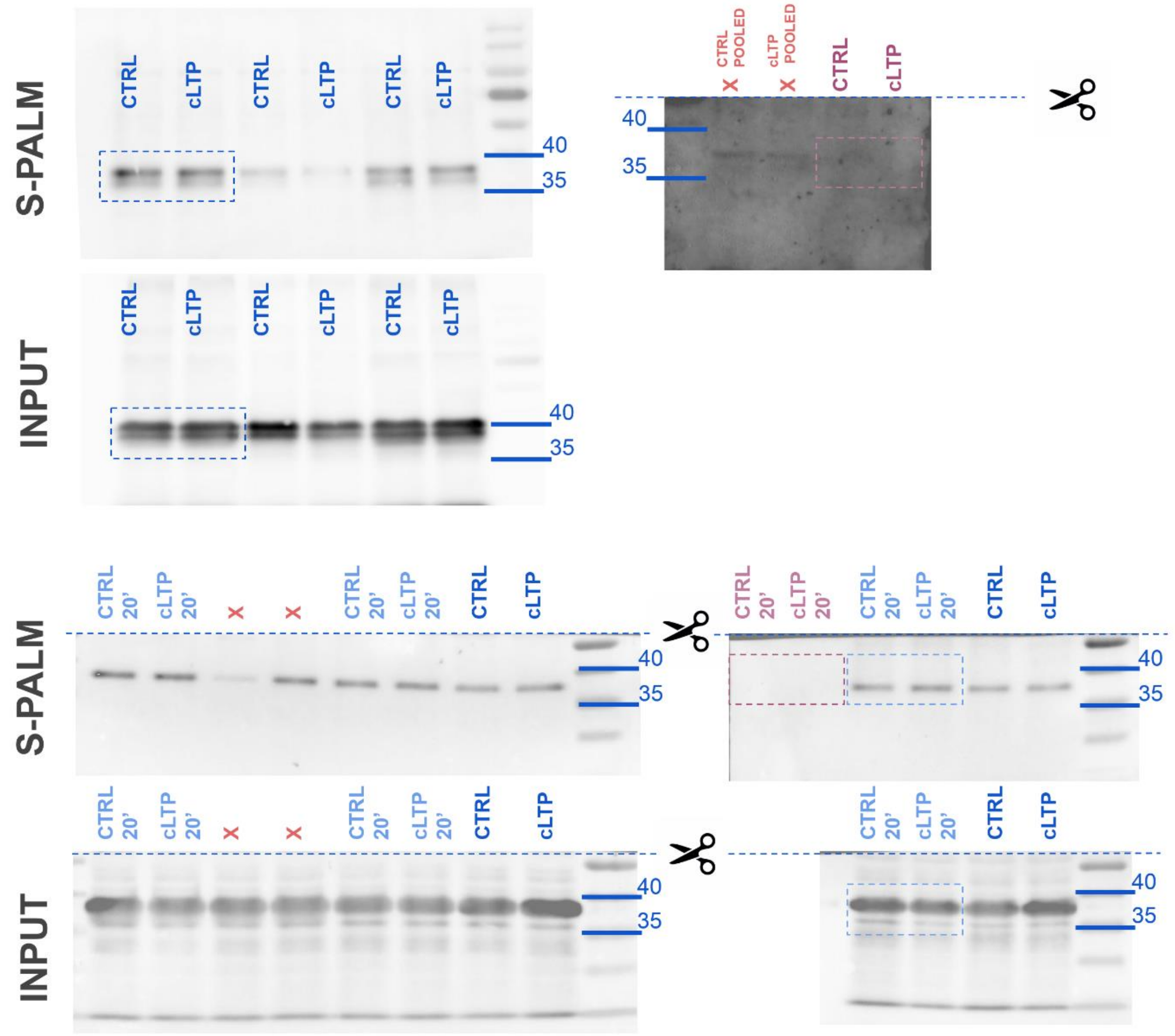

ABE RESULTS

NEURONAL CULTURES

FIG. 1D

positive results (HAM+)
negative controls (HAM-)
× irrelevant lanes (not included in the results)
replicate shown in the main figure

VAMP2

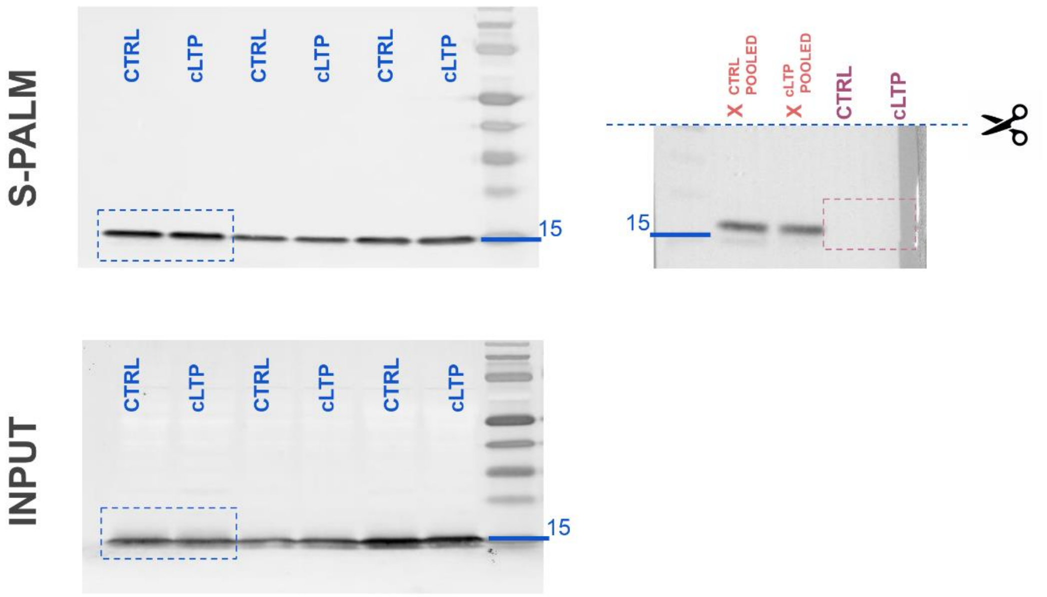

SNAP25

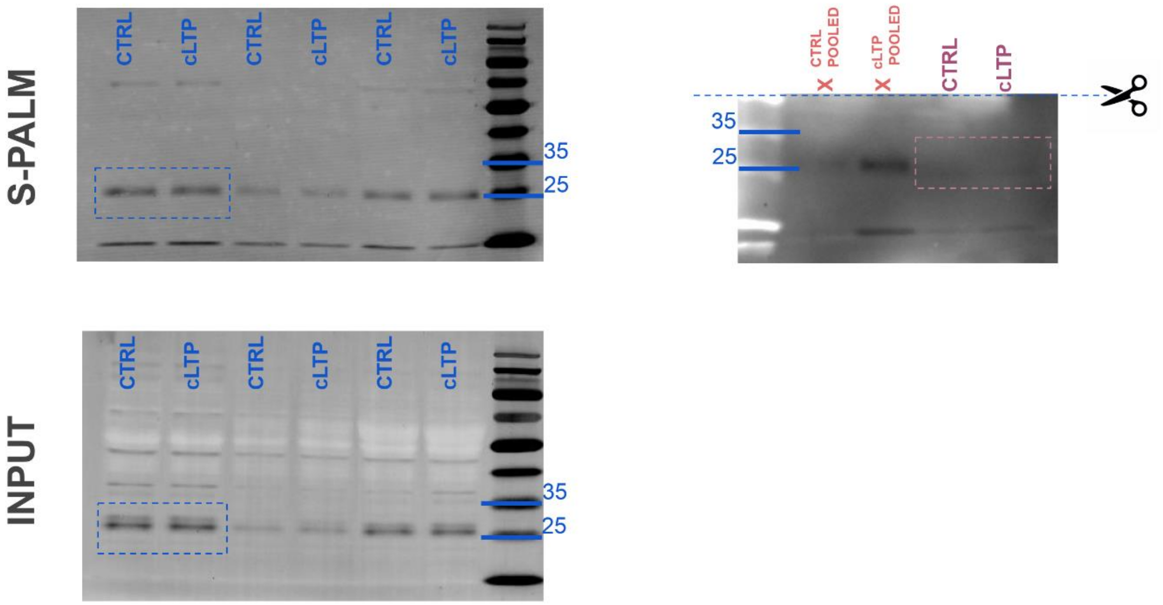

ABE RESULTS

NEURONAL CULTURES

FIG. 1E AND FIG. S1E

positive results (HAM+)
negative controls (HAM-)
× irrelevant lanes (not included in the results)
replicate shown in the main figure

PSD95

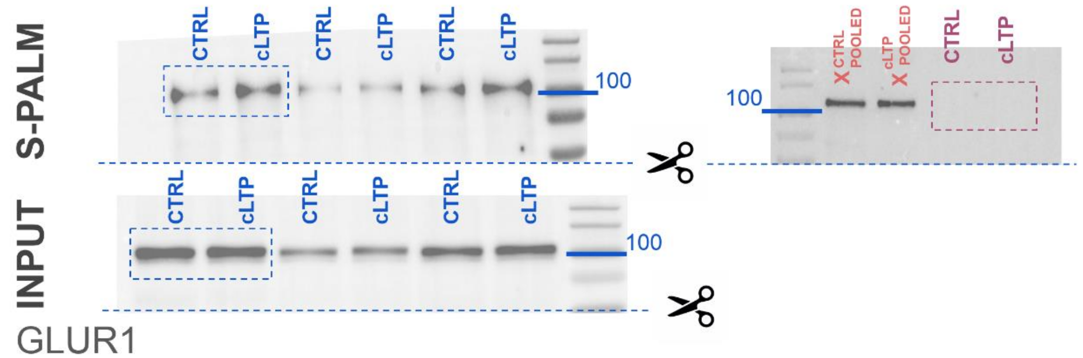

GLUR1

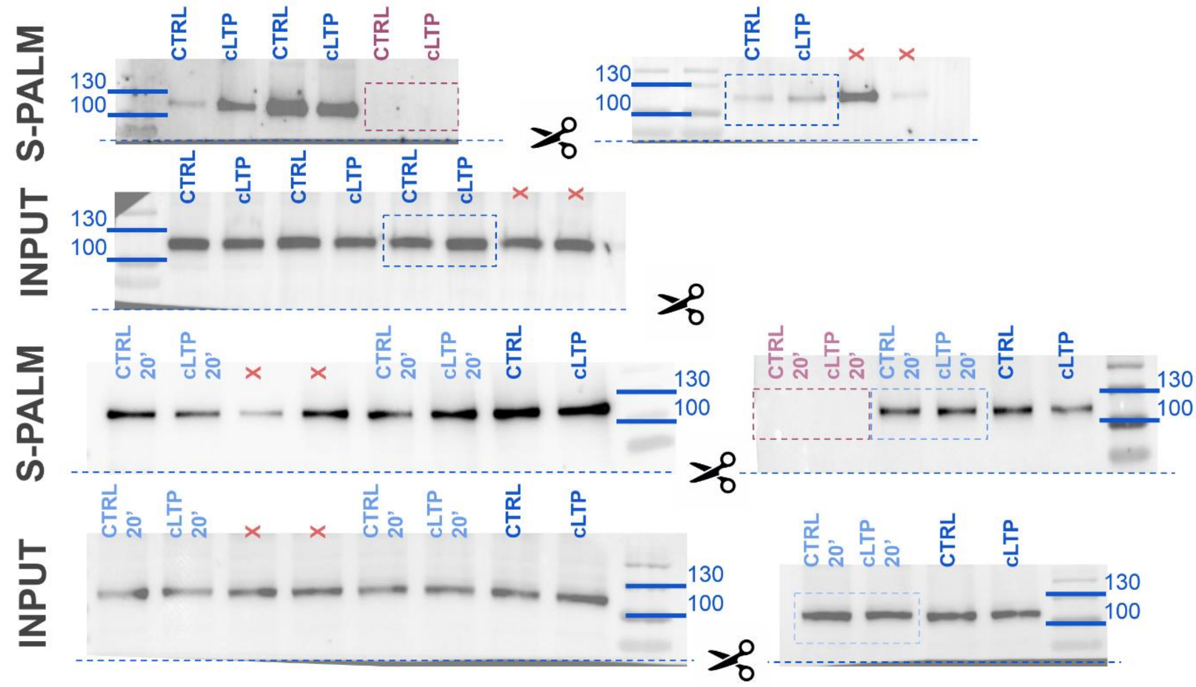

NEUROCHONDRIN

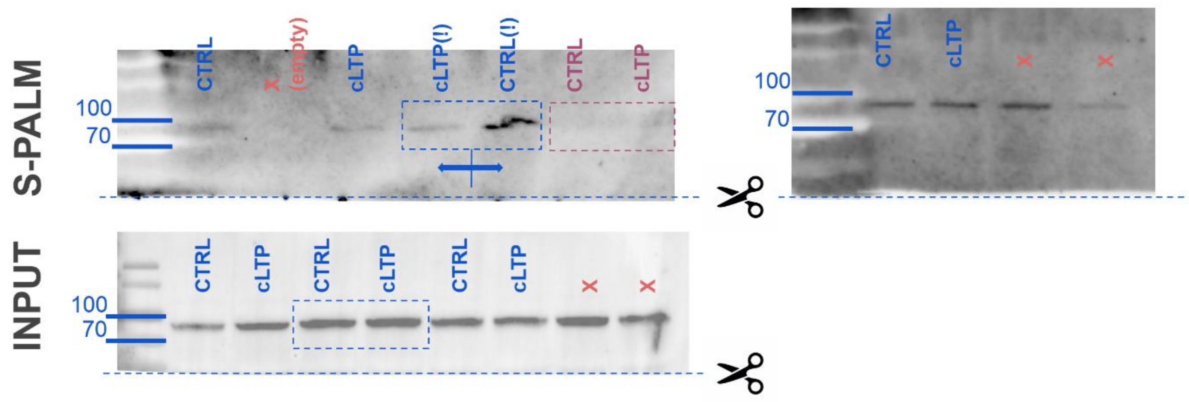

ABE RESULTS

NEURONAL CULTURES

FIG. 1D AND FIG. S1D

NCAM

positive results (HAM+)
negative controls (HAM-)
× irrelevant lanes (not included in the results)
replicate shown in the main figure

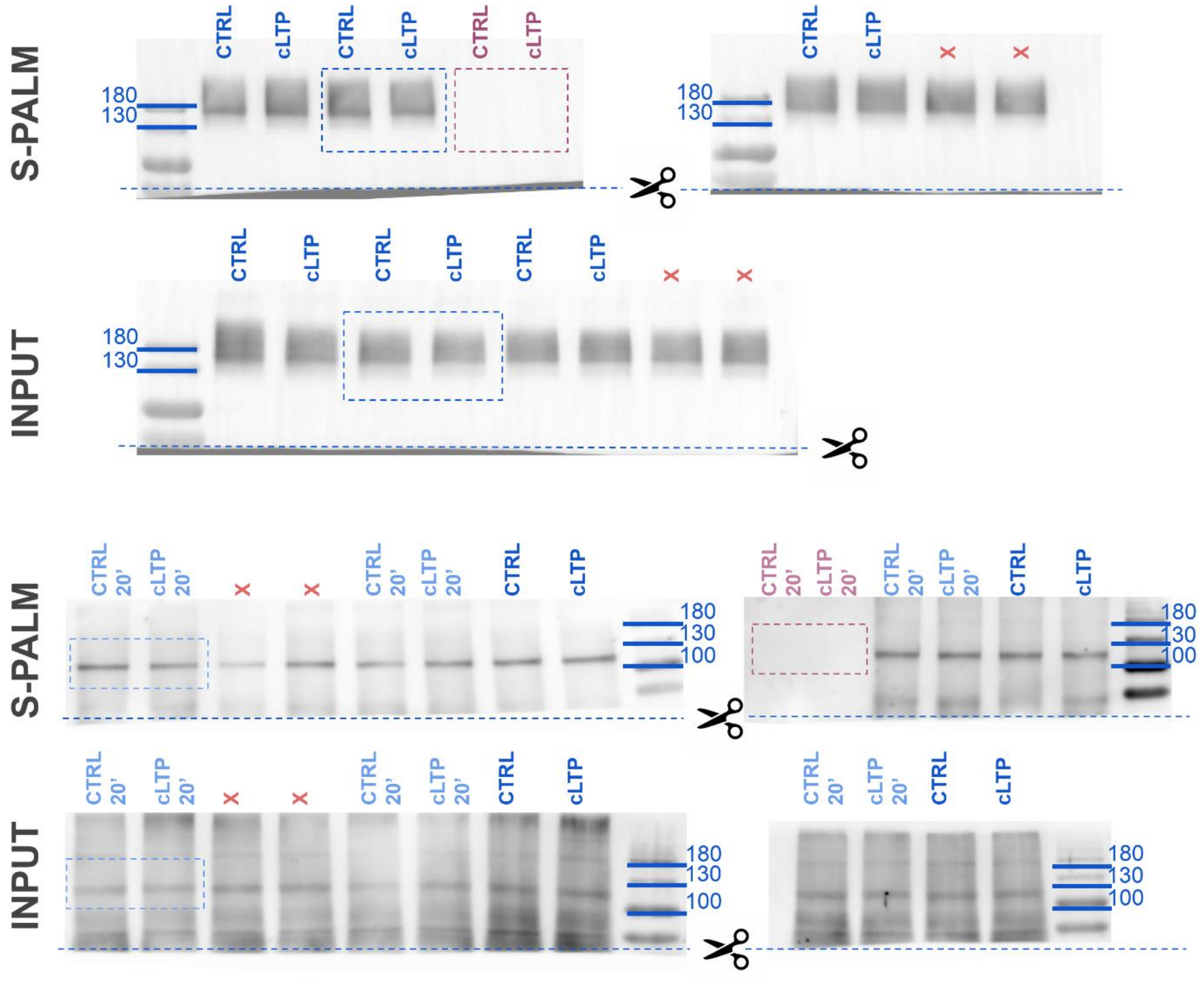

ABE RESULTS

HIPPOCAMPAL SLICES

FIG. 3B

replicate shown in the main figure

Arc

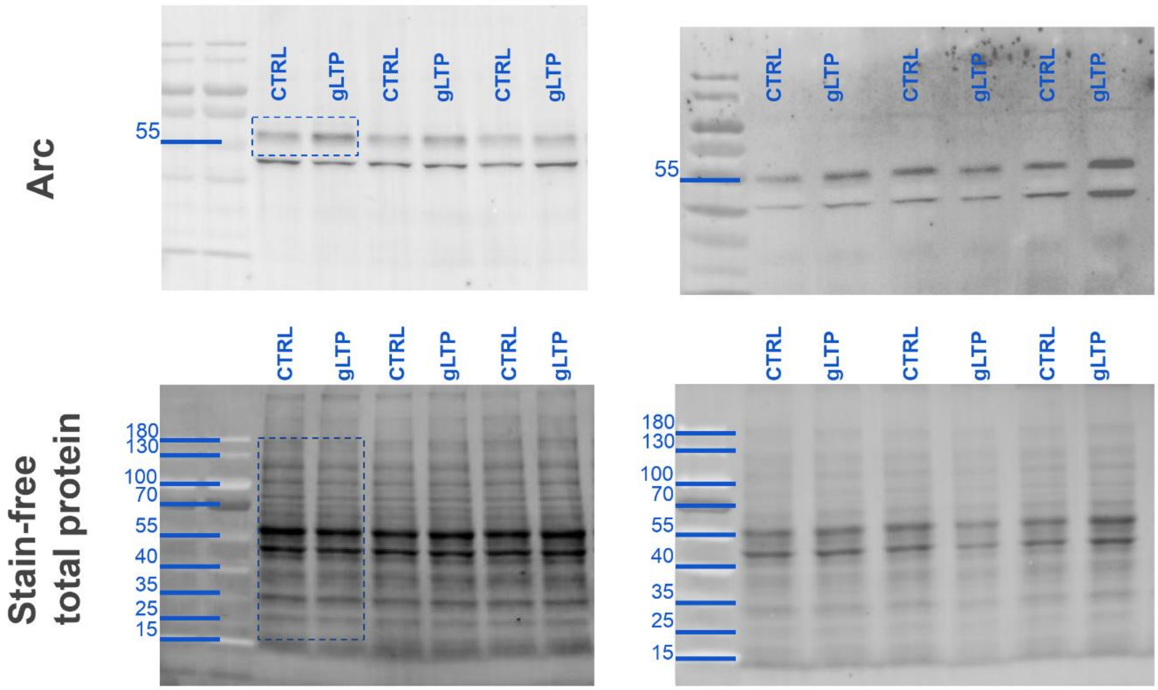

ABE RESULTS

HIPPOCAMPAL SLICES

FIG. 3D

positive results (HAM+)
negative controls (HAM-)
× irrelevant lanes (not included in the results)
replicate shown in the main figure

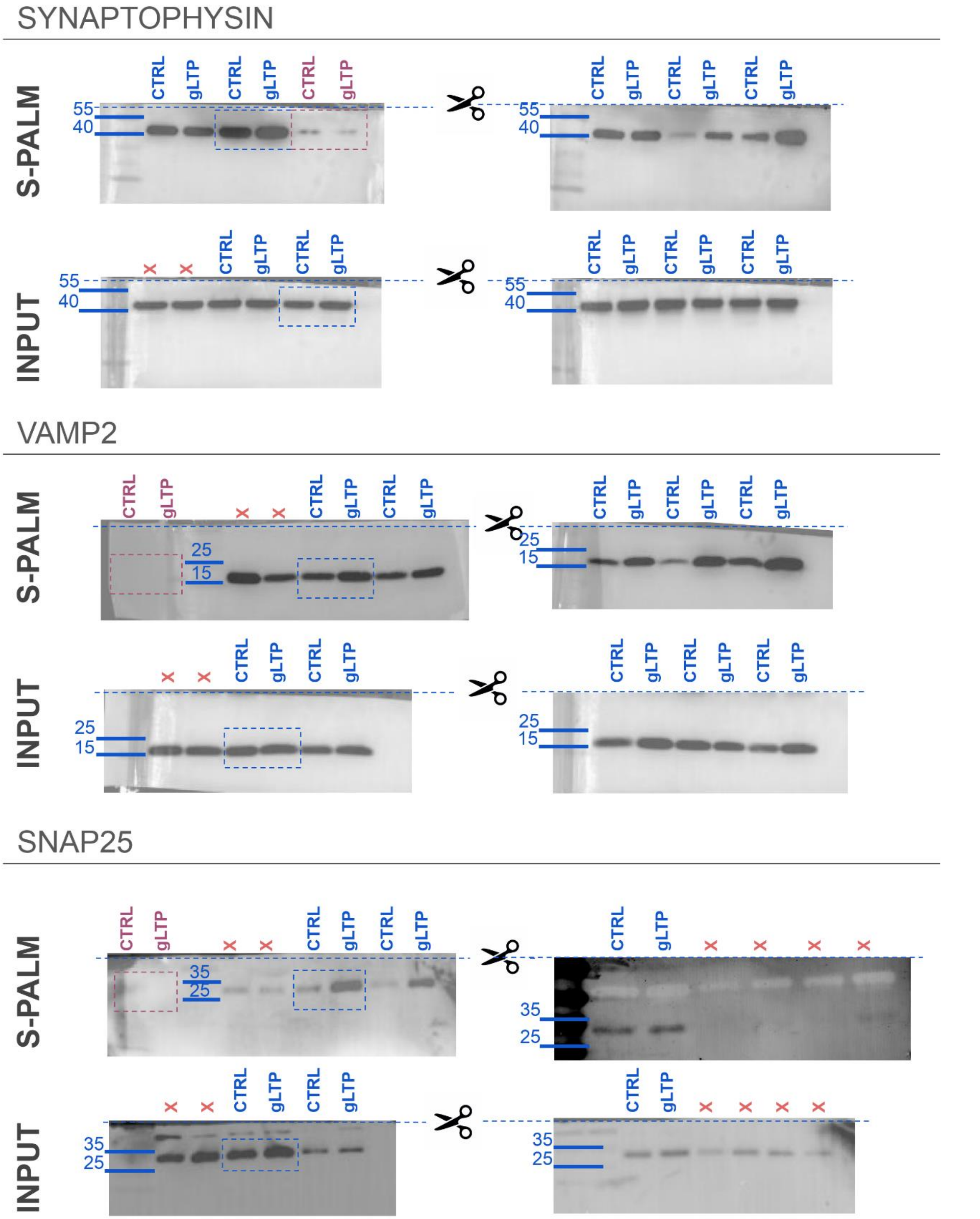

ABE RESULTS

HIPPOCAMPAL SLICES

FIG. 3E

positive results (HAM+)
negative controls (HAM-)
× irrelevant lanes (not included in the results)
replicate shown in the main figure

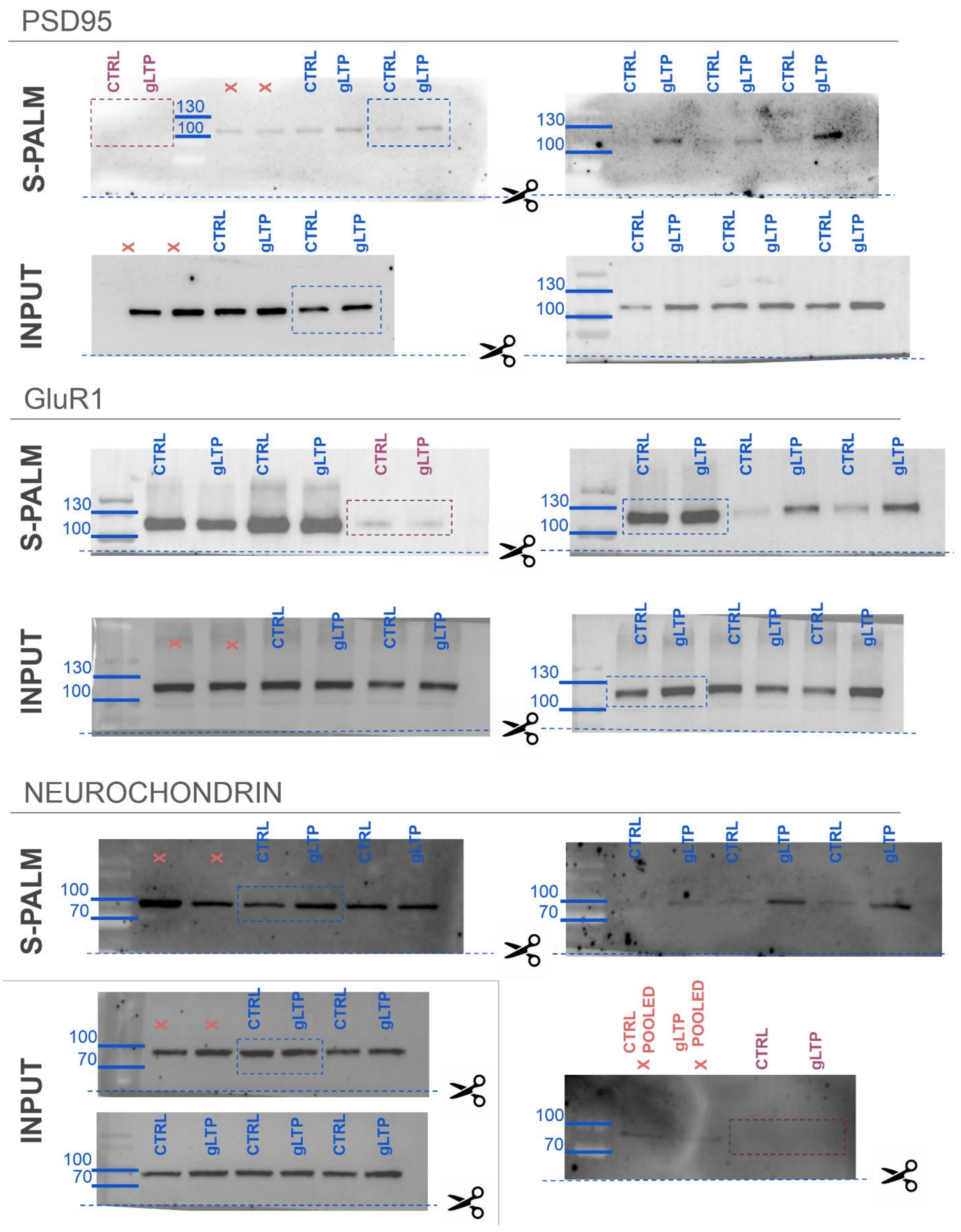

ABE RESULTS

HIPPOCAMPAL SLICES

FIG. 3F

NCAM

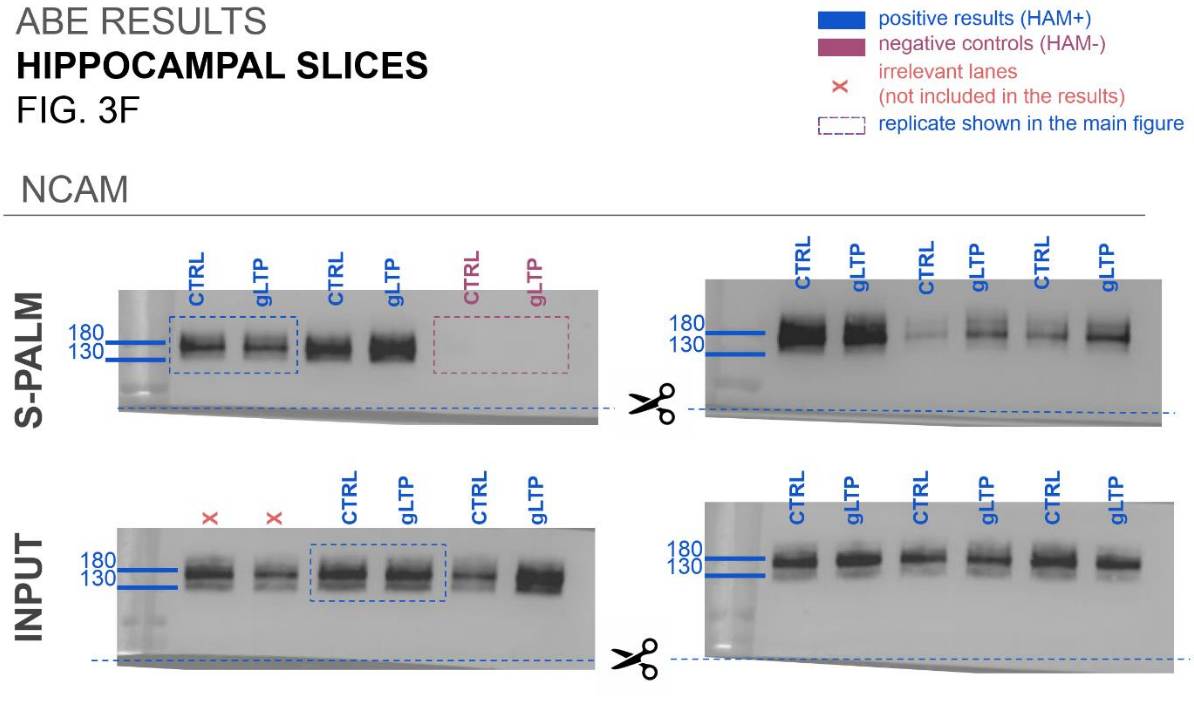

ABE RESULTS

SYNAPTONEUROSOMES

FIG. 5B

replicate shown in the main figure

pERK / ERK

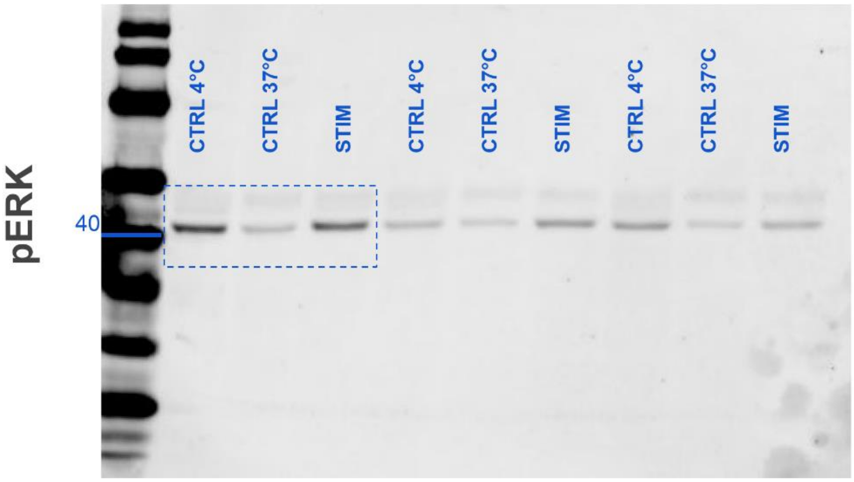

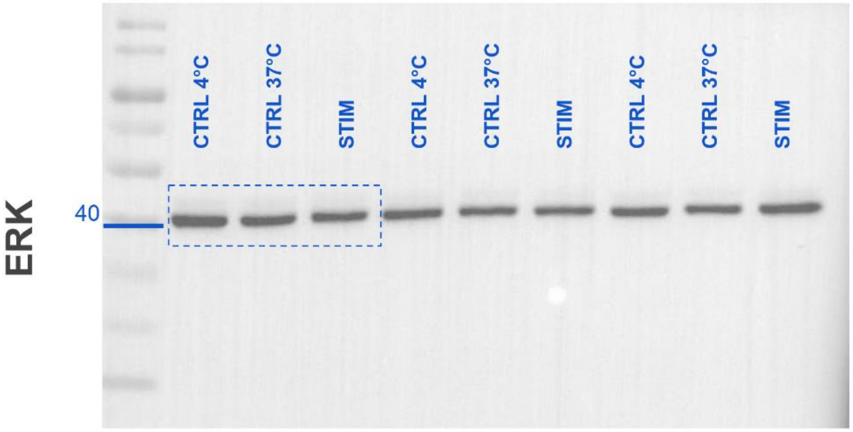

ABE RESULTS

SYNAPTONEUROSOMES

FIG. 5D

positive results (HAM+)
negative controls (HAM-)
× irrelevant lanes (not included in the results)
replicate shown in the main figure

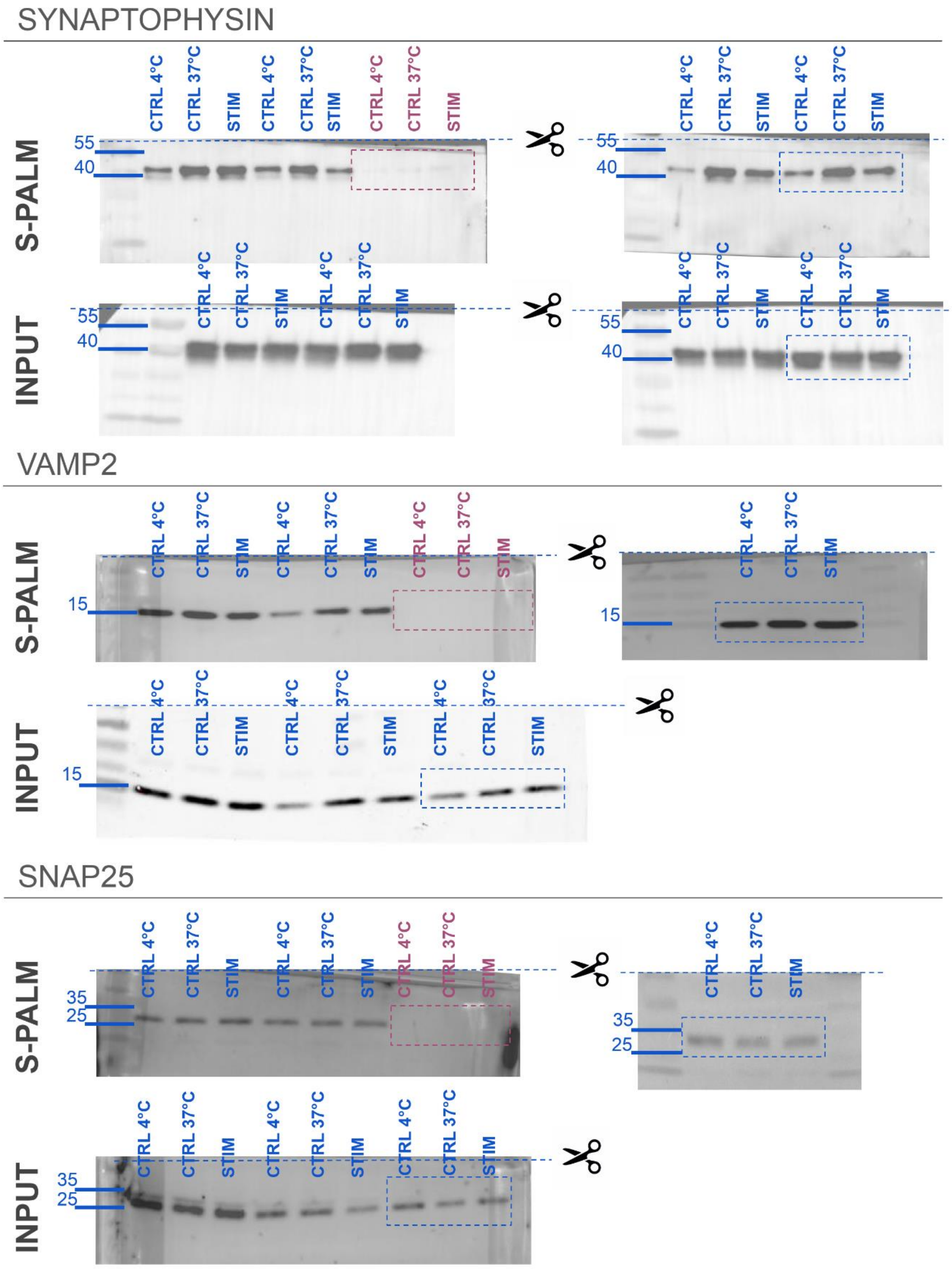

ABE RESULTS

SYNAPTONEUROSOMES

FIG. 5E AND 5F

positive results (HAM+)
negative controls (HAM-)
irrelevant lanes (not included in the results)
replicate shown in the main figure

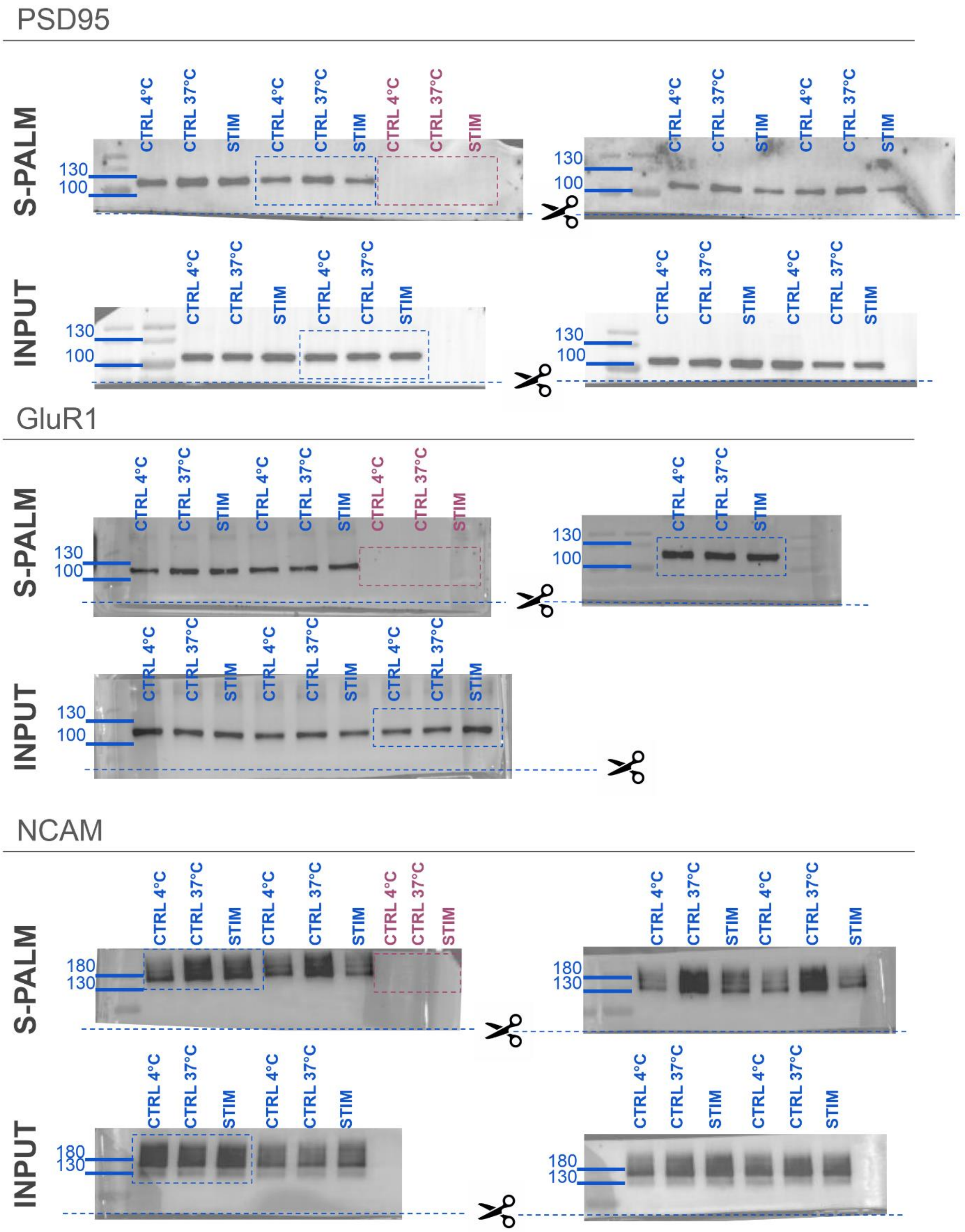

ABE RESULTS

SYNAPTONEUROSOMES

FIG. S6

replicate shown in the main figure

PSD95

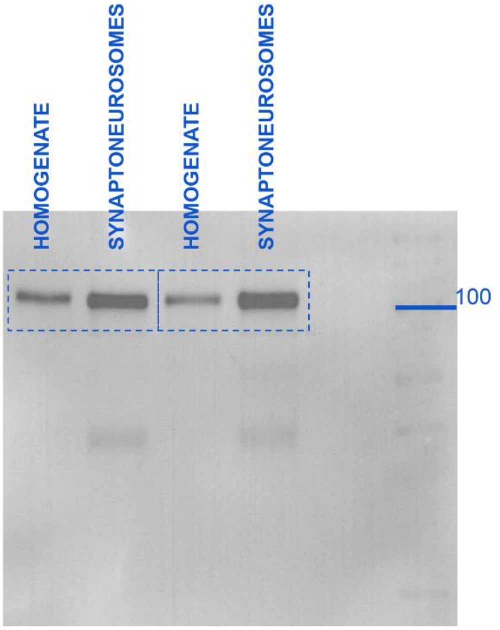

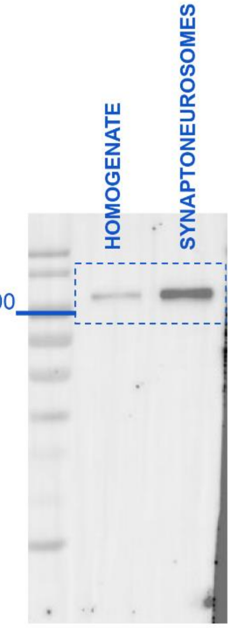

GEPHYRIN

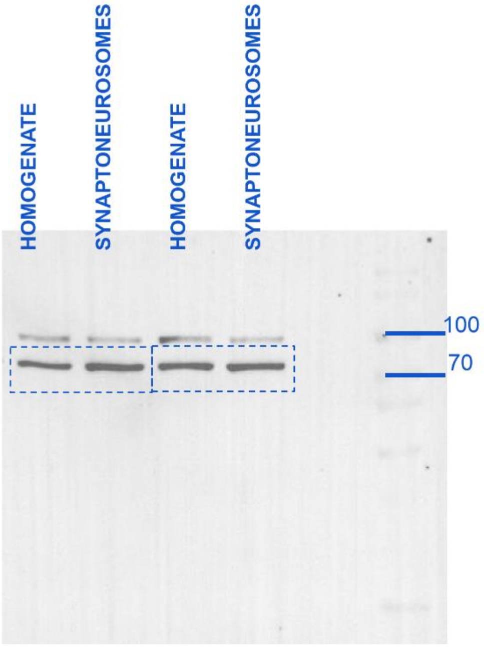

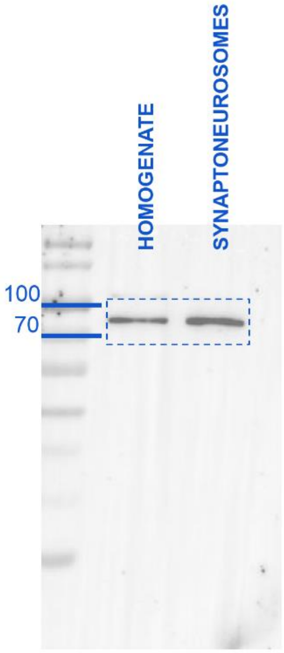

Data S1.

Palmitoyl-proteins from this proteomic study of synaptoneurosomes.

The Excel spreadsheet 1 contains a list of all input proteins. Sheet 2 shows a list of all palmitoylated proteins. Sheet 3 shows differentially palmitoylated proteins detected in STIM 37ºC (when compared to CTR 37ºC samples) or in CTR 37ºC (when compared to CTR 4ºC samples).
